# Early Findings on Functional Connectivity Correlates of Behavioral Outcomes of Brain-Computer Interface Stroke Rehabilitation Using Machine Learning

**DOI:** 10.3389/fnins.2018.00624

**Published:** 2018-09-11

**Authors:** Rosaleena Mohanty, Anita M. Sinha, Alexander B. Remsik, Keith C. Dodd, Brittany M. Young, Tyler Jacobson, Matthew McMillan, Jaclyn Thoma, Hemali Advani, Veena A. Nair, Theresa J. Kang, Kristin Caldera, Dorothy F. Edwards, Justin C. Williams, Vivek Prabhakaran

**Affiliations:** ^1^Department of Radiology, University of Wisconsin-Madison, Madison, WI, United States; ^2^Department of Electrical Engineering, University of Wisconsin-Madison, Madison, WI, United States; ^3^Department of Biomedical Engineering, University of Wisconsin-Madison, Madison, WI, United States; ^4^Department of Kinesiology, University of Wisconsin-Madison, Madison, WI, United States; ^5^Medical Scientist Training Program, University of Wisconsin–Madison, Madison, WI, United States; ^6^Neuroscience Training Program, University of Wisconsin–Madison, Madison, WI, United States; ^7^Department of Psychology, University of Wisconsin–Madison, Madison, WI, United States; ^8^Department of Orthopedics and Rehabilitation, University of Wisconsin-Madison, Madison, WI, United States; ^9^Department of Medical Physics, University of Wisconsin-Madison, Madison, WI, United States

**Keywords:** brain-computer interface, stroke recovery, functional connectivity, motor impairment, machine learning, support vector regression

## Abstract

The primary goal of this work was to apply data-driven machine learning regression to assess if resting state functional connectivity (rs-FC) could estimate measures of behavioral domains in stroke subjects who completed brain-computer interface (BCI) intervention for motor rehabilitation. The study cohort consisted of 20 chronic-stage stroke subjects exhibiting persistent upper-extremity motor deficits who received the intervention using a closed-loop neurofeedback BCI device. Over the course of this intervention, resting state functional MRI scans were collected at four distinct time points: namely, pre-intervention, mid-intervention, post-intervention and 1-month after completion of intervention. Behavioral assessments were administered outside the scanner at each time-point to collect objective measures such as the Action Research Arm Test, Nine-Hole Peg Test, and Barthel Index as well as subjective measures including the Stroke Impact Scale. The present analysis focused on neuroplasticity and behavioral outcomes measured across pre-intervention, post-intervention and 1-month post-intervention to study immediate and carry-over effects. Rs-FC, changes in rs-FC within the motor network and the behavioral measures at preceding stages were used as input features and behavioral measures and associated changes at succeeding stages were used as outcomes for machine-learning-based support vector regression (SVR) models. Potential clinical confounding factors such as age, gender, lesion hemisphere, and stroke severity were included as additional features in each of the regression models. Sequential forward feature selection procedure narrowed the search for important correlates. Behavioral outcomes at preceding time-points outperformed rs-FC-based correlates. Rs-FC and changes associated with bilateral primary motor areas were found to be important correlates of across several behavioral outcomes and were stable upon inclusion of clinical variables as well. NIH Stroke Scale and motor impairment severity were the most influential clinical variables. Comparatively, linear SVR models aided in evaluation of contribution of individual correlates and seed regions while non-linear SVR models achieved higher performance in prediction of behavioral outcomes.

## Introduction

### Brain computer interface

Electroencephalogram (EEG)-based brain-computer interface (BCI) technology has emerged as a therapeutic modality for stroke rehabilitation that has been demonstrated to facilitate additional recovery that conventional therapies have not been able to accomplish thus far (Silvoni et al., 2011). EEG-based BCI detects and uses a patient's neural signals as inputs to provide real-time feedback, effectively enabling users to modulate their brain activity. This is a promising intervention for patients with motor impairment, as they can control external devices such as computers and robots during rehabilitative tasks without relying on residual muscle control (Felton et al., 2009) which could be tailored to individuals potentially yielding greater benefits from the system (Bhagat et al., 2016). Specifically, EEG-based BCI intervention using attempted movement with functional electrical stimulation (FES) (Biasiucci et al., 2018) and tongue stimulation (TS) enables us to detect intent-to-move brain signals and provide users with both visual and tactile sensory feedback as a form of reward for producing certain brain activity patterns while performing specific tasks. Thus far, several neuroimaging studies in the realm of stroke rehabilitation have shown potential functional benefits associated with the use of BCI technology including, but not limited to, modulating changes in neuroplasticity and restoring motor function (Várkuti et al., 2013; Young et al., 2014c; Nair et al., 2015; Soekadar et al., 2015).

### Functional magnetic resonance imaging

In recent years, neuroimaging has become integral in studying the progression in neurodegenerative processes and efficacy of rehabilitation procedures (Caria et al., 2011; Song et al., 2014; Young et al., 2014c; Nair et al., 2015). Task-free methods such as resting state functional magnetic resonance imaging (rs-fMRI) allow us to measure the temporal correlation of the spontaneous, low-frequency (<0.1 Hz) blood-oxygen-level-dependent (BOLD) signals across distinct brain regions at rest. Oscillations in these BOLD fMRI signals are believed to reflect cortical dynamic self-organization and have been associated with the neural reorganization underlying cognitive and motor function during stroke recovery (Lee et al., 2013; Bajaj et al., 2015). Additionally, recent neuroimaging studies have demonstrated overlap among networks identified during rs-fMRI, motor imagery fMRI tasks, and motor execution fMRI tasks (Grefkes et al., 2008; Nair et al., 2015). The motor network is a complex and highly dynamic system with a unique balance of excitatory and inhibitory mechanisms which has been postulated to be significantly disturbed after the event of stroke (Grefkes and Fink, 2011). This specific neuronal network commonly includes the primary motor area (M1), premotor cortex (PMC) and supplementary motor area (SMA), as it is established that activity in these cortical regions maintains a dynamic equilibrium at resting-state and is modulated during task performance (Debaere et al., 2001). Recently, we have demonstrated that changes in task-related brain connectivity can be used as a diagnostic tool to track cortical changes and behavioral outcomes following BCI intervention in patients with stroke (Young et al., 2014c). However, while there is evidence of overlap among resting-state and motor-related fMRI task (Grefkes et al., 2008), these resting state networks have yet to be completely characterized in the context of motor recovery facilitated by the use of a BCI device. Therefore, further investigation into changes in resting-state connectivity in relation to changes in associated behavioral function following BCI intervention is necessary.

### Multivariate data analysis

The ability of data-driven machine learning techniques to model multivariate relationships can be attributed to their application in neuroimaging analysis. Several studies have shed light on the utility of machine learning to perform classification tasks (Dai et al., 2012; Meier et al., 2012; Rehme et al., 2014; Fergus et al., 2016; Khazaee et al., 2016; Ding et al., 2017; Mohanty et al., 2018). These advance our understanding of brain function by identifying brain patterns associated with specific neurological diseases and differentiating among patient groups. However, performing simple binary classification might not suffice to answer clinically relevant questions such as prediction of recovery associated with neuropathological disease and time until onset of specific disease-related symptoms. In comparison to classification-based studies, relatively fewer studies have examined neuroimaging data from the perspective of prediction of outcomes (Dosenbach et al., 2010; Vergun et al., 2013) using machine learning approaches. This underscores the need to use data modeling techniques that can predict outcomes on a more continuous scale while handling the high dimensionality of input data. Within machine learning, there exist a variety of algorithms to perform real-valued outcome prediction such as naïve Bayesian (Frank et al., 2000), k-nearest neighbors (Hastie and Tibshirani, 1996), Gaussian process (Marquand et al., 2010) regression models. Rapid developments in the field are utilizing neural networks (Pereira et al., 2016) in large datasets. However, in this work we focus on using the a support vector machine-based regression model which is proficient in modeling linear as well as non-linear relationships between variables with a modest sample size and present an extension of the work previously presented (Mohanty et al., 2017). In place of relying solely on non-linear models, we compared their performance to the linear case, which enabled us to pinpoint specific correlates of behavioral outcomes and improve interpretability for future clinical applications. Additionally, the relative contribution of individual seed regions was analyzed, and comparative analysis helped establish the trade-off involved in choosing one model over the other.

### Overview of this study

In the realm of stroke rehabilitation research, there have been concerted efforts focusing on evaluating the neurophysiological changes post-stroke (Rossini et al., 2003; Teasell et al., 2005; Kwakkel et al., 2008; Wang et al., 2010) and investigating novel therapeutic interventions to promote motor recovery and ultimately improve overall quality of life for patients (Levy et al., 2001; Kwakkel et al., 2008; Young et al., 2014d). While EEG-based BCI intervention has shown early promise as a form of rehabilitation post-stroke, neuroplastic changes in the form of functional connectivity and resulting therapeutic effects on behavioral outcomes following this intervention coupled with FES and TS remain to be elucidated. In this study, correlates of behavioral measures and associated changes following this EEG-based BCI intervention are investigated using brain connectivity as well as behavioral measures at preceding stages. Resting-state functional connectivity (rs-FC) was examined in previously identified (Grefkes et al., 2008) motor network comprised of eight seed regions that play a dominant role in motor initiation, specification, and execution. Immediate as well as carry-over effects were investigated by examining fMRI and behavioral measures at three stages: prior to the start of intervention, upon completion of intervention and 1-month post completion of intervention. To this end, a multivariate regression scheme, based on support vector machines, was employed to handle the multi-dimensional data and examine utility in estimating individual behavioral outcomes and associated changes. The purpose of this study was four-fold: (i) to identify neural correlates based on rs-FC within the motor network to estimate behavioral outcomes following BCI intervention; (ii) to identify neural correlates based on changes in rs-FC within the motor network to estimate changes in behavioral measures following the BCI intervention; (iii) to identify behavioral correlates at a preceding time-point to estimate behavioral measures at a succeeding time-point; and (iv) to study the impact of potential confounds relative to rs-FC and behavior as correlates of behavioral outcomes following the intervention.

## Materials and methods

### Study design

**Figure 1:**
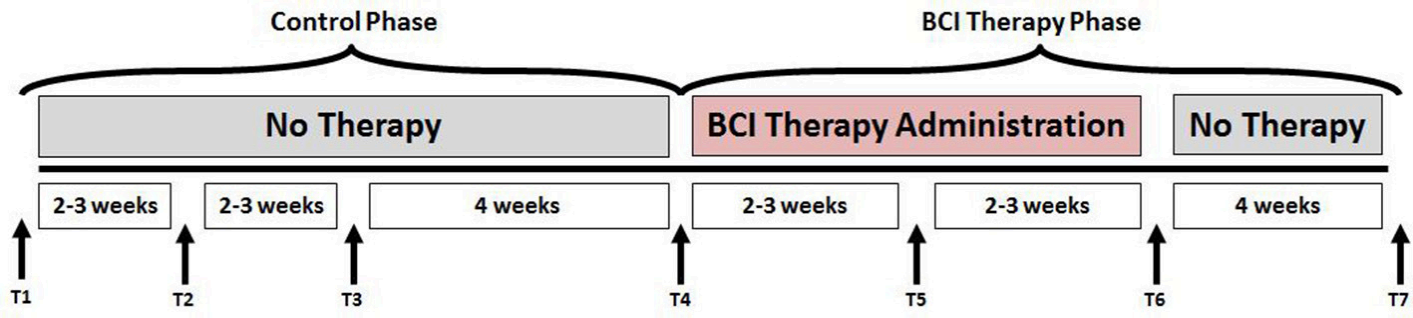
Study paradigm. The time-points at which neuroimaging and behavioral data were collected are represented by - T1: Control baseline 1, T2: Control baseline 2, T3: Control baseline 3, T4: Intervention baseline T5: Mid-intervention, T6: Post-intervention, and T7: 1-month post-intervention.

This study followed a permuted-block design that accounted for gender, stroke chronicity, and severity of motor impairment in stroke subjects to randomly assign subjects to one of two groups: crossover control group or BCI therapy (intervention) group. The study paradigm is schematized in Figure 1. Subjects in the BCI therapy group received this intervention and were administered a battery of behavioral assessments and MRI scans at four time-points throughout the intervention: pre-intervention (T4), mid-intervention (T5), immediately post-intervention (T6), and 1-month after completing the last BCI intervention session (T7). Subjects in the crossover control group first received three functional assessments and MRI scans during the control phase in which no BCI intervention was administered (T1 through T3), and their assessments were spaced at intervals similar to those given during the BCI intervention phase. Upon completion of the control phase of the study, the crossover control group “crossed over” into the BCI therapy phase of the study. In this study, neuroimaging and behavioral data corresponding to pre-intervention, post-intervention and 1-month post-intervention time-points across the crossover control and the BCI intervention groups were combined and treated as a single sample group to provide additional power to the analysis.

### Participants

Subjects for this analysis were recruited as part of an ongoing multi-arm stroke rehabilitation study intended to evaluate the effects of intervention using an EEG-based BCI device on the recovery of upper-extremity motor function. The inclusion criteria for participation in the study were: (1) at least 18 years of age; (2) persistent upper-extremity motor impairment resulting from an ischemic or hemorrhagic stroke; (3) ability to provide written informed consent. Exclusion criteria for the study consisted of: (1) concomitant neurodegenerative or other neurological disorders; (2) psychiatric disorders or cognitive deficits that would preclude a subject's ability to provide informed consent; (3) pregnant or likely to become pregnant during the study; (4) allergies to electrode gel, metal and/or surgical tape, contraindications to MRI; (5) concurrent treatment for infectious disease. The study was approved by the Health Sciences Institutional Review Board of University of Wisconsin-Madison. Written informed consent was obtained from all subjects prior to the start of their participation in the study. Twenty chronic stroke subjects (10 from crossover control group and 10 from BCI intervention group), who completed the BCI intervention, were included in this analysis. We limited the cohort for this study to chronic-stage (time since stroke onset >6 months) stroke subjects only. Excluding stroke subjects in the acute (time since stroke onset <14 days) and sub-acute (time since stroke onset <6 months) stages was critical for this analysis to ensure that spontaneous recovery in these stages does not confound the effects of the BCI intervention. In other words, changes observed in both rs-FC and motor behavioral performance during the acute and sub-acute phases might result from spontaneous neuroplasticity processes rather than from the BCI intervention. Time since stroke was defined to be the period between stroke onset and baseline visit. In addition, subjects were excluded from this analysis if they exhibited bilateral brain lesions for the potential reason that they could be outliers and confound the results. All neuroimaging scans were inspected by a neuroradiologist for the purposes of lesion localization. The distribution of lesion site in the cohort was as follows: middle cerebral artery territory (MCA; *N* = 10), frontal lobe (*N* = 3), cerebellum (*N* = 2), putamen (*N* = 2), occipital lobe (*N* = 1), basal ganglia (*N* = 1), and internal carotid artery occlusion (*N* = 1). Stroke severity was determined by NIH Stroke Scale (NIHSS) (Brott et al., 1989) scores at baseline. Severity of motor impairment was assessed based on performance on Action Research Arm Test (Carroll, 1965; Lang et al., 2006) and visual inspection at the preliminary visit. Participants' handedness post-stroke was established before the start of intervention based on Edinburgh Handedness Inventory (Oldfield, 1971). Participant characteristics are summarized in Table 1.

**Table 1:** Demographic and clinical characteristics of the study cohort.

Number of stroke subjects	20
Chronicity	Chronic (>6 months since stroke onset)
Age (mean ± std. dev in years)	62.4 ± 14.27
Gender	8 Females, 12 Males
Lesion hemisphere	8 Left, 12 Right
Stroke severity (mean NIHSS ± std. dev)	3.75 ± 3.5
Motor impairment severity	11 Severe, 9 Moderate
Time since stroke (mean ± std. dev in months)	37.65 ± 40.84
Post-stroke handedness	16 Right, 2 Left, 2 Ambidextrous

### BCI intervention

All participants received at least 9 and up to 15 two-hour EEG-based BCI interventional sessions, with up to three sessions per week; the complete intervention lasted up to 6 weeks The BCI intervention was administered using BCI2000 software (Schalk et al., 2004) with modifications for administering TS (TDU 01.30, Wicab Inc.) and FES (LG-7500, LGMedSupply; Arduino 1.0.4). EEG signals, which served as the input for the BCI device, were detected and recorded from a 16-channel EEG cap and amplifier (Guger Technologies) during intervention.

A brief account of the three-step intervention is provided as follows. (i) Each intervention session began with an open-loop calibration screening task in which subjects were instructed to attempt movement of either their left or right hand with resting periods in-between by following randomly ordered visual cues on the screen, such as “Right,” “Left,” or “Rest,” in 4-s blocks. During the initial screening session, participants did not receive any form of feedback. The EEG activity, recorded in the open-loop screening task, was used by the classifier for identifying activation patterns corresponding to volitional movement of the respective left and right hands in the closed-loop task. Both in the initial screening and closed-loop feedback conditions, attempted movement was utilized to simulate the training conditions of the neurofeedback task similar to the cognitive processes involved in real-world movement. (ii) Following the initial screening, subjects performed a closed-loop task, in which they received real-time visual feedback in the context of a cursor task game. The goal of the cursor task game was to move a cursor (ball) onto a target area, with target areas positioned on either the left or right side of the computer screen. Subjects were instructed to move their left or right hand to control the corresponding movement of the cursor in the direction of the target on the screen. A 70% accuracy was set as the criteria to establish control of a BCI system in this phase (Kübler et al., 2001, 2005). Real-time EEG signals were used to calculate and control lateral cursor movement, which served as the visual feedback for the remainder of the session. During each BCI intervention session, subjects completed 10 runs of this game, which included 8–12 trials per run, while receiving continuous visual feedback. (iii) After successful completion of 10 runs of the game with visual feedback, both TS and FES were simultaneously incorporated into the intervention session for the remaining trials (as many trials as possible within a 2-h session). FES, with a pulse rate of stimulation 60 Hz and varied up to 5 mA in increments of 0.5 mA as per the participant's comfort level, was administered to muscles of the subject's impaired forearm when their neural activity signals corresponding to impaired arm movement intent were detected during a trial in which subjects attempted to move the cursor to a target on the screen corresponding to the side of the impaired arm. The stimulation thresholds for FES and TS were determined during the first intervention session and maintained at the same level in all the subsequent sessions for consistency. This EEG-based BCI system with FES and TS provides subjects with both visual and tactile sensory feedback. To keep subjects engaged throughout the task, the size of the target on the screen and speed of the cursor could be changed to modulate the difficulty of the task depending on their accuracy. Additional details of the procedure of the intervention can be found in prior studies such as those described by Wilson et al. (2009), and Young et al. (2014a,c).

### Neuroimaging data acquisition

Neuroimaging data were acquired at the four aforementioned time points (T4 through T7). For the purposes of this work, we chose to use the data from three of these points, i.e., prior to starting the intervention or pre-intervention assessment (T4), immediately upon completion of intervention or post-intervention assessment (T6) and a month after completion of full intervention (T7) to study the potential peak and carry-over effects of the EEG-based BCI intervention. Rs-fMRI scans were acquired on GE 750 3T MRI scanners (GE Healthcare, Waukesha, WI) using an 8-channel head coil. Ten-minute resting state scans were acquired while participants' eyes were closed using single-shot echo-planar T2^*^-weighted imaging: *TR* = 2600 ms, 231 time-points, TE = 22 ms, FOV = 224 mm, 64 × 64 matrix size, flip angle = 60°, and 40 slices with voxel dimensions of 3.5 × 3.5 × 3.5 mm^3^. Five-minute T1-weighted anatomical images were obtained at the start of each scan using a BRAVO FSPGR sequence with the following parameters: TR = 8.16 ms, TE = 3.18 ms and TI = 450, matrix size = 256 × 256, 156 slices, flip angle = 12°, FOV = 256 mm with slice thickness = 1 mm.

### Behavioral assessments

To assess the behavioral impact of the BCI intervention, a battery of objective and subjective measures was administered to participants at each time-point. Corresponding to the neuroimaging, we focused on behavioral measures at pre-intervention (T4), post-intervention (T6) and 1-month post-intervention (T7) in this study. To systematically quantify motor functional outcomes, the following standard behavioral measures were evaluated as summarized in Table 2: the Action Research Arm Test (ARAT) (Carroll, 1965; Lang et al., 2006), 9-Hole Peg Test (9HPT) (Chen et al., 2009), Barthel Index (BI) (Mahoney, 1965), and Stroke Impact Scale (SIS) (Duncan et al., 1999; Carod-Artal et al., 2008). The ARAT serves as a standardized and reliable functional measure for stroke rehabilitation that measures changes in specific upper limb function among individuals who sustained cortical damage resulting in hemiplegia. The 9HPT measure is used for quantifying hand dexterity. ARAT and 9HPT were observed for the affected [ARAT(A), 9HPT(A)] as well as unaffected [ARAT(U), 9HPT(U)] upper extremity. In this study, BI was administered in questionnaire form and not observed from functional performance as it was originally designed and validated. The BI score quantifies the ability of an individual to care for her/himself in their daily life. The SIS scores are self-reported outcomes that measure the health status of stroke subjects. SIS includes the following standard domains: Activities of Daily Living (ADL) for difficulty carrying out activities in a typical day, Hand Function (HF) for difficulty in using the hand most affected by stroke, Mobility (Mob) for difficulty in ability to be mobile at home and in community, and Physical Strength (PS) for overall strength in the upper and lower limbs of the affected side.

**Table 2:** Summary of all the behavioral assessments used as outcomes.

**Behavioral assessment**	**Category**
ARAT(U): Action Research Arm Test for the upper extremity unaffected by stroke	Objective
ARAT(A): Action Research Arm Test for the upper extremity affected by stroke	Objective
9HPT(U): 9-Hole Peg Test for the upper extremity unaffected by stroke	Objective
9HPT(A): 9-Hole Peg Test for the upper extremity affected by stroke	Objective
BI: Barthel Index	Objective
SIS(ADL): Activities of daily life domain of Stroke Impact Scale	Subjective
SIS (HF): Hand function domain of Stroke Impact Scale	Subjective
SIS(Mob): Mobility domain of Stroke Impact Scale	Subjective
SIS(PS): Physical strength domain of Stroke Impact Scale	Subjective

### Individual level analysis

**Figure 2:**
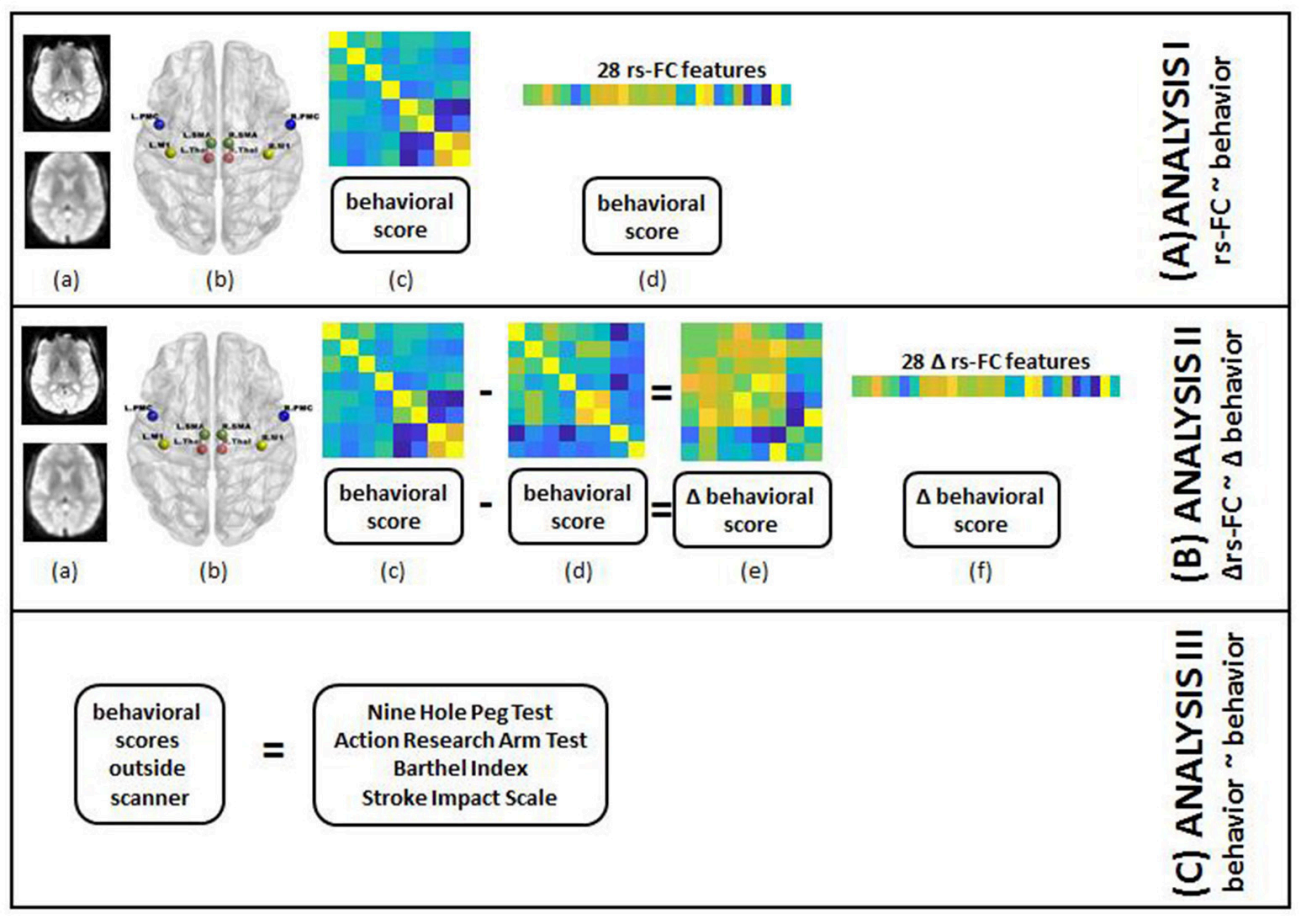
Steps for individual subject analysis are shown below. **(A) rs-FC correlates of behavior:** (a) raw rs-fMRI (top) from pre-, post- and 1-month post-interventions were preprocessed (bottom); (b) 8 seed regions were chosen from the motor network to compute rs-FC at each time-point; (c) 8 × 8 rs-FC matrix was computed and corresponding behavioral scores were transformed as needed for each time-point; (d) rs-FC reflected in the lower triangle of 8 × 8 matrix was vectorized into 28 unique correlation coefficients per subject and 8 distinct behavioral measures were aggregated for group-level analysis. **(B) Δrs-FC correlates of Δ behavior:** (a) raw rs-fMRI (top) from pre-, post- and 1-month post-interventions were preprocessed (bottom); (b) 8 seed regions were chosen from the motor network to compute rs-FC at each time-point; (c) 8 × 8 rs-FC matrix was computed and corresponding behavioral scores were transformed as needed for a preceding time-point; (d) 8 × 8 rs-FC matrix was computed and corresponding behavioral scores were transformed as needed for a succeeding time-point; (e) change in rs-FC and behavioral scores were calculated between the two time-points; (f) change in rs-FC reflected in the lower triangle of 8 × 8 matrix was vectorized into 28 unique correlation coefficients per subject and change in 8 distinct behavioral measures were aggregated for group-level analysis. **(C) behavioral correlates at preceding time-point of behavior at succeeding time-point:** transformed scores for 8 behavioral measures at pre-, post- and 1-month post-interventions were aggregated for group-level analysis.

The main steps involved in the processing of data on a single-subject level are outlined in Figure 2 and described in detail in the following subsections.

#### Neuroimaging preprocessing

Rs-fMRI scans of 20 subjects were visually inspected for artifacts and preprocessed in the following sequential manner: the first three volumes of each scan were removed, images were despiked, slice time corrected, aligned with the corresponding anatomical T1 scan, spatially smoothed with a 4-mm FWHM (full width at half maximum) Gaussian kernel, transformed into the standard MNI space (3.5 mm isotropic), motion censored (per TR motion > 1 mm or 1°), regressed for nuisance variables (regressed out the signal from locally averaged white matter and cerebrospinal fluid) and bandpass filtered (0.009–0.08 Hz). Given the controversial nature of global signal regression (Murphy and Fox, 2016), this processing step was not included in the analysis pipeline. All rs-fMRI data were preprocessed using Analysis of Functional NeuroImages (AFNI) (http://afni.nimh.nih.gov/afni) (Cox, 1996).

#### Rs-FC

**Figure 3:**
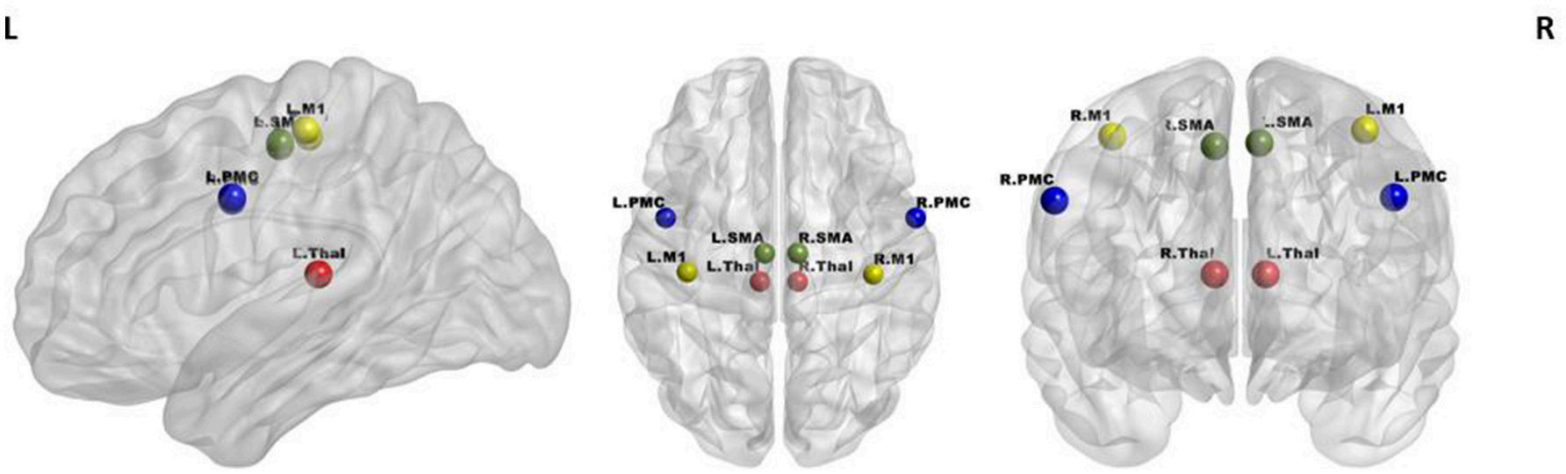
Regions of interest in the motor network included four bilateral seeds: M1 (yellow), PMC (blue), SMA (green), and Thalamus (red).

A seed-based analysis was adopted based on prior work that investigated rs-FC within the motor network in stroke population (Grefkes et al., 2008; Nair et al., 2015). The seed regions were identified on the basis of a network of cortical and subcortical areas that exhibited activation during visually paced hand movements. The seed regions for this study included the primary motor cortex (M1), supplementary motor area (SMA), thalamus, and lateral premotor cortex (PMC) in the right and left hemispheres, as illustrated in Figure 3 using BrainNet Viewer (Xia et al., 2013) and abbreviated as per Table 3. The MNI coordinates, also specified in Table 3, for the eight regions were used to create 8-mm spherical seeds. For each subject, BOLD time series signal from each region was extracted from the spatially standardized residuals obtained in the preprocessing stage. The extracted time series for each region was used to compute an 8 × 8 ROI correlation matrix for each subject. From this symmetric matrix, 28 unique correlation coefficients were extracted to represent pairwise rs-FC within the motor network at each of the three stages of interest.

**Table 3:** Shorthand representation of the eight ROIs in the motor network used for the analysis is presented below.

**ROI**	**Shorthand**	**X (MNI)**	**Y (MNI)**	**Z (MNI)**
Left primary motor cortex	L.M1	−39	−22	57
Right primary motor cortex	R.M1	40	−23	55
Left premotor cortex	L.PMC	−48	1	36
Right premotor cortex	R.PMC	58	1	35
Left supplementary motor area	L.SMA	−6	−14	53
Right supplementary motor area	R.SMA	8	−14	52
Left thalamus	L.Thal	−8	−26	12
Right thalamus	R.Thal	8	−26	12

#### Potential clinical confounds

The study cohort was heterogeneous with respect to multiple clinical factors which could confound the contribution of rs-FC alone. Based on prior studies, we identified the following factors as potential confounds: age and stroke severity (Ferraro et al., 2003), severity of motor impairment, and time since stroke (Rehme et al., 2012), lesion hemisphere (Crinion et al., 2007), and gender (Kelly-Hayes et al., 2003). We included these clinical variables as features, built the regression model for each outcome, and compared the performances of models with and without the confounding variables. This strategy would help understand the impact of potential confounds on the performance of regression model as well as the contribution of confounds as correlates relative to rs-FC or behavioral features.

### Group-level analysis

Applications of machine learning regression models such as SVR on rs-fMRI have been demonstrated in neuroimaging-based studies (Dosenbach et al., 2010; Vergun et al., 2013) as SVR-based methods can efficiently handle multi-dimensional data and model the linearity as well as non-linearity in a given dataset. For the purposes of this study, we adopt a strategy, similar to these studies. To understand the correlates of behavioral outcomes and changes, the following analyses were undertaken by applying SVR to correlate:

**ANALYSIS I:** rs-FC at preceding time-points with behavioral outcomes at succeeding time-points (T4 with T6; T4 with T7; T6 with T7).
**ANALYSIS II:** change (Δ) in rs-FC between pairs of time-points with corresponding change (Δ) in behavioral outcomes (T4 and T6; T4 and T7; T6, and T7).
**ANALYSIS III:** behavioral measures at preceding time-points with behavioral measures at succeeding time-points (T4 with T6; T4 with T7; T6 with T7).

In case of behavioral measures, total scores across comprising domains for BI and ARAT, average scores across two trials for 9HPT, and transformed scores to yield a percentage of possible points for the SIS domains of PS, Mob, HF, and ADL were considered.

To characterize changes among the three stages of interest (T4, T6 and T7), the following definitions were employed:

(1) $$\begin{array}{r} \Delta rs-FC=\frac{rs-{FC}_{succeeding\;stage}-rs-{FC}_{preceding\;stage}}{rs-{FC}_{preceding\;stage}} \end{array}$$

where *rs*−*FC*_*succeeding stage*_ and *rs*−*FC*_*preceding stage*_ denote the values of *rs*−*FC* correlation at succeeding (T6, T7) and preceding (T4, T6) stages respectively.

Unlike in case of Δ*rs*−*FC*, the definition for changes in behavioral measures differed by case. For 9HPT(A), 9HPT(U), ARAT(U), BI, SIS (PS, Mob, and ADL) scales, the normalized change was gauged by:

(2) $$\begin{array}{r} \Delta behavior=\frac{{behavior}_{succeeding\;stage}-{behavior}_{preceding\;stage}}{{behavior}_{preceding\;stage}} \end{array}$$

However, in case of ARAT(A) and SIS(HF), the possibility of *behavior*_*precedingstage*_ being 0 invalidates the above normalization. Thus, for these two outcomes, a simple deviation was computed as follows:

(3) $$\begin{array}{r} \Delta behavior={behavior}_{succeeding\;stage}-{behavior}_{preceding\;stage} \end{array}$$

where *behavior*_*succeedingstage*_ and *behavior*_*precedingstage*_ correspond to the scores achieved by a participant in each behavioral task at succeeding (T6, T7) and preceding (T4, T6) stages respectively. Due to lack of variability across most time-points, the ARAT(U) was discarded as a behavioral outcome for all analyses.

Each of the three aforementioned analyses was examined by including the identified potential confounding variables as well. In each case, the input features for all subjects were aggregated and the steps described as follows were implemented.

#### Feature selection

Each regression model was built using a subset of input features (28 rs-FC features, 28 Δ rs-FC features and 8 behavioral measures as described by ANALYSES I, II and III) through a feature selection procedure. A forward sequential feature selection (SFS) was helpful in reducing the dimensions of the original data for better interpretation of features involved (He et al., 2013; Lu et al., 2015). This method searches for a subset of features that optimally models a given outcome. The algorithm adds each candidate feature and checks the specified criteria by building a regression model based on selected features. The criteria specified for selection of a feature involved minimization of the mean squared error (MSE) arising from estimation error for SVR model. The SVR model is described in the following section. A nested leave-one out cross-validation approach allowed for testing of estimation error on the left-out sample, where the inner loop was used to choose the features during a training-validation phase. One advantage of methods such as SFS is that since it works in the raw feature space, it can be applied to both continuous and categorical features. During cross-validation, the features that were common across all the folds were reported as the contributing features for each model. The weights assigned to these features were averaged across all folds and sorted to determine the rank or importance of individual features in the regression model.

#### Support vector regression (SVR)

Once a subset of features was selected by SFS, the SVR model was trained using the selected features for each behavioral outcome. SVR was chosen due to its ability to predict real valued behavioral outcomes based on multi-dimensional input features using the principle of supervised learning support vector machines (SVM) (Scholkopf and Smola, 2001). Typically used as a classifier, SVM can also be used for regression analysis (Vapnik, 2013). SVR forms a non-parametric method via the kernel trick. This method not only provides resilience to overfitting and good generalization performance, but also helps in interpreting the contribution of individual features in high-dimensional data with a linear kernel. The principle behind using the SVR analysis is described in Supplementary Section 1. In the case of linear regression, the mapping function lies in the input space, so it is possible to derive the weights corresponding to each input feature. However, in the case of non-linear regression, similar weights cannot be derived explicitly since the mapping function is no longer found in the input space but in the feature space in the kernel space. Both linear and non-linear kernel SVR models were employed for our analyses.

#### Cross-validation

A leave-one-out cross-validation (LOOCV) approach (Hastie et al., 2001) was adopted to estimate the performance of the regression model in the outer loop of the nested cross-validation as it provides an approximation of the test error with a lower bias and is more suitable for a dataset with a limited number of samples such as that used in this analysis. We performed a LOOCV by subject in this validation-testing phase. This means that the data consisting of 20 observations were subdivided into 20 folds such that each fold comprised of data from a single subject. The regression model was trained using selected features from 19 folds and tested upon the left-out fold. This was repeated 20 times such that data from each subject was left out once while a model was trained using the rest of the data. The performance of the model was quantified in terms of the average root-mean-squared error (RMSE) for linear and non-linear SVR over all iterations of LOOCV given by:

(4) $$\begin{array}{r} RMSE=\sqrt{\frac{1}{l}\overset{l}{\underset{i=1}{\sum }}{\left(y_{est_{i}}-y_{i}\right)}^{2}} \end{array}$$

where the *y*_*es*_*t*__*i*__−*y*_*i*_ term is the measure of error between the estimated outcome and the true outcome. Reasonable performance of SVR is characterized by values of RMSE closer to 0. In addition to RMSE, the linear SVR can also be assessed in terms of goodness of fit in terms of the coefficient of determination (R^2^). However, it is not an appropriate measure for non-linear models as illustrated by simulations performed by Spiess and Neumeyer (2010). Thus, we quantified performance of linear SVR models by R^2^ and RMSE but compared linear and non-linear models in terms of RMSE.

#### Model parameter optimization

The generalization performance is dependent upon both the selected features and model parameters *C*, ε (Burges, 1998; Smola and Schölkopf, 2004), and the kernel parameters. The parameter *C* is used to trade-off between the complexity of the model and the extent to which estimated deviations larger than ε are tolerated in formulation of the optimization. Parameter ε controls the width of the ε -insensitive zone, used to fit the training data. Both *C*, ε values have an impact on complexity of the model. The data points are scaled by the parameter depending upon the kernel used for regression. A randomized search method based on Bayesian optimization process attempts to minimize the MSE in the separate LOOCV by varying the parameters for 30 evaluations (Bull, 2011; Snoek et al., 2012; Gelbart et al., 2014) which corresponded to the inner loop of the training-validation phase, training on all samples but one with the best chosen parameters and testing on the left out sample.

#### Evaluation of regression model

In order to validate the results against chance levels, non-parametric permutation tests were performed. For each regression model, the outcome labels were randomly permuted 1,000 times and feature selection and LOOCV were repeated for each permuted dataset to create a null distribution. The performance of the regression model corresponding to the non-permuted data was considered significantly better than chance if the RMSE of the model was lower than at least 95% of those obtained from the null-hypothesis.

### Overview of methodology

**Figure 4:**
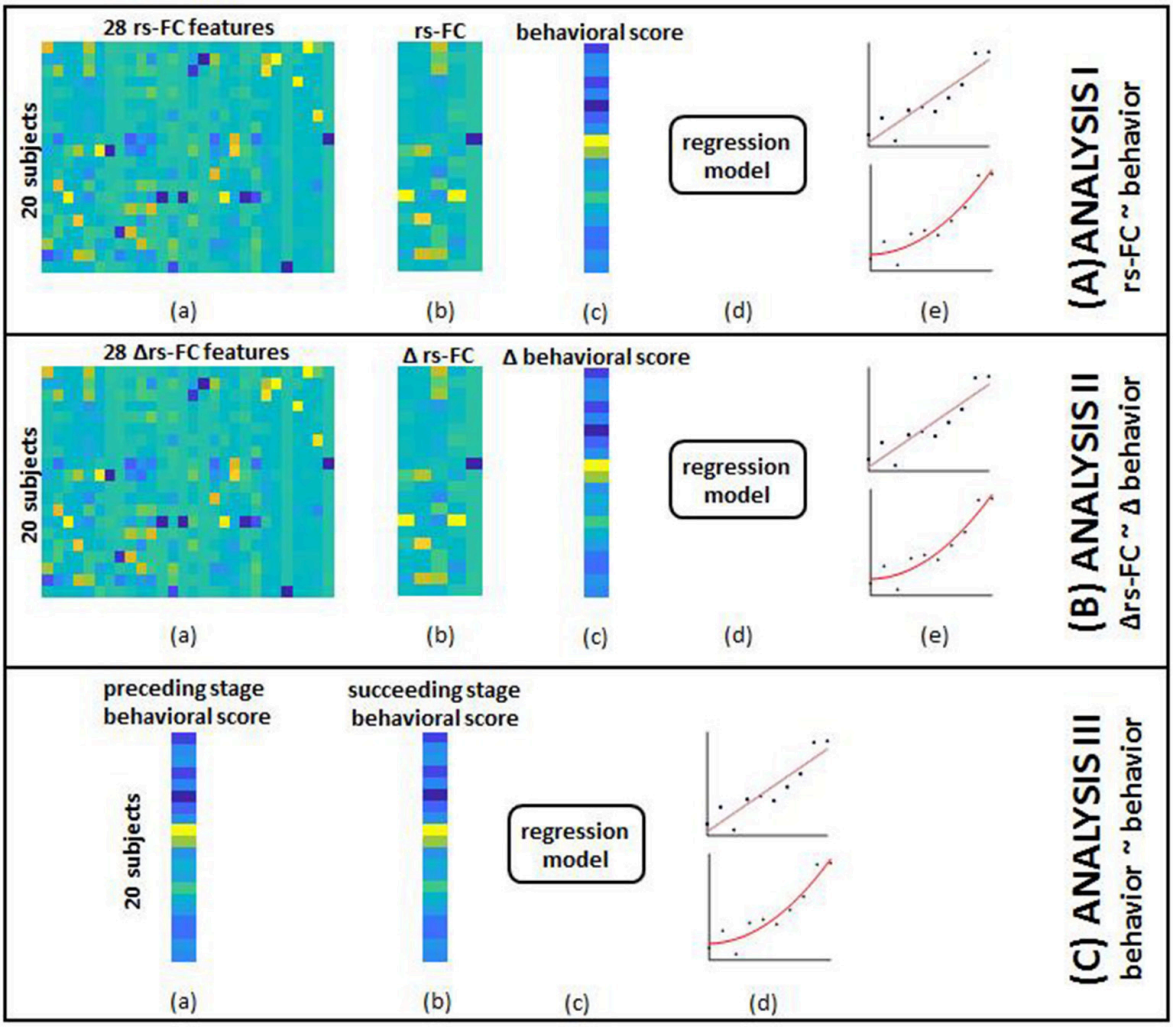
The overview of group-level analysis is provided here. **(A) rs-FC correlates of behavior:** (a) aggregated data from single-subject analysis gave 28 rs-FC features for each of the 20 subjects; (b) SFS was used to select specific correlates corresponding to each behavioral outcome; (c) aggregated behavioral scores for 20 subjects served as outcomes in separate models; (d) data from (b) and (c) were fed into the SVR model; (e) linear (top) and non-linear (bottom) kernels were specified to perform regression. Steps (a through e) were repeated by adding identified clinical variables to rs-FC data as input features. **(B) Δrs-FC correlates of Δbehavior:** (a) aggregated data from single-subject analysis gave 28 change in rs-FC features for each of the 20 subjects between pairs of time-points; (b) SFS was used to select specific correlates corresponding to each behavioral outcome; (c) aggregated change in behavioral scores between corresponding pair of time-points for 20 subjects served as outcomes in separate models; (d) data from (b) and (c) were fed into the SVR model; (e) linear (top) and non-linear (bottom) kernels were specified to perform regression. Steps (a–e) were repeated by adding identified clinical variables to change in rs-FC data as input features. **(C) behavioral correlates at preceding time-point of behavior at succeeding time-point:** (a) aggregated behavioral scores from a preceding time point gave 8 distinct measures; (b) aggregated behavioral scores from a succeeding time-point gave the corresponding 8 measures; (c) data from steps (a) and (b) were fed to the SVR model; (d) linear (top) and non-linear (bottom) kernels were specified to perform regression.

Overall, we trained SVR models using selected rs-FC, Δrs-FC, or behavioral measures, optimized the model and identified the contributing input features that provided the minimum RMSE upon LOOCV. All computations were carried out using the Statistics and Machine Learning Toolbox in MATLAB R2017a (The MathWorks, Inc., Natick, Massachusetts, United States). The group-level pipeline of analysis is visualized in Figure 4.

## Results

We present the findings from the linear-kernel SVR here (results corresponding to the non-linear kernel models can be found in Supplementary Materials ST1–3).

### Choice of time-points of interest

The analyses, undertaken here, revolved around three time-points, namely T4, T6, and T7, i.e., pre-intervention, post-intervention and 1-month post-intervention. The objective was to study the immediate as well as potential residual impact of the intervention after a month. A comparison of group medians of behavioral outcomes at these three time-points, evident from **SF 1**, showed increased values at T7 relative to T4 or T6 for SIS(Mob), SIS(HF), ARAT(A) although not significant (based on a Mann Whitney U-test). The time-points from the control period, i.e., T1 through T3 were not included in the regression analyses due to limited samples (*N* = 10). However, we did not find significant differences (using Mann Whitney U-test on each pair of time-points) when the group medians of the behavioral outcomes during the control period were compared with T4 as illustrated in **SF 2**. Thus, presumably, we could consider measures at T4 to serve as representative scores for the control period.

### Performance of correlates

Behavioral outcomes were estimated using rs-FC, Δrs-FC as well as behavioral measures at preceding time-points. In terms of R^2^, better estimation of outcomes was observed using behavioral correlates, followed by rs-FC and Δrs-FC in order. This held true with and without the impact of clinical variables.

#### Rs-FC as correlates of behavioral outcomes

The performances of SVR using rs-FC as correlates of behavioral outcomes are presented in Table 4 (and **ST 1**). All the SVR models, developed here, performed better than chance-level based on permutation test (*p* < 0.05) as depicted in **SF3** of Supplementary Section 2. Individual predictors involved in estimating the different outcomes are listed in Table 5 (and **ST 4)**. Overall, rs-FC associated with L.M1, R.M1, and R.PMC were the main contributors toward estimation, both with and without clinical variables. Among the three time-points, better performances were found in cases of correlating rs-FC at T6 and behavioral measures at T7.

**Table 4:** Linear-kernel SVR performances based on leave-one out cross-validation to correlate rs-FC at preceding time-point with behavioral measures at succeeding time-point are presented.

**Outcome**	**T4 rs-FC~T6 behavior**			**T4 rs-FC~T7 behavior**			**T6 rs-FC~T7 behavior**		
	**Features**	**RMSE**	**R^**2**^**	**Features**	**RMSE**	**R^**2**^**	**Features**	**RMSE**	**R^**2**^**
**(A) WITHOUT CLINICAL VARIABLES**									
9HPT(A)	9	110.93^*^	0.21	14	116.69^*^	0.14	6	109.25^*^	0.25
9HPT(U)	5	4.2^*^	0.27	3	4.05^*^	0.26	6	2.86^*^	0.63
ARAT(A)	2	20.58^*^	0.33	6	17.87^*^	0.49	4	15.48^*^	0.61
BI	3	8.01^*^	0.24	5	6.31^*^	0.31	11	6.39^*^	0.3
SIS(ADL)	4	10.93^*^	0.14	4	10.62^*^	0.51	5	109.25^*^	0.25
SIS(HF)	4	31.81^*^	0.37	6	26.84^*^	0.36	5	33.47^*^	0.64
SIS(Mob)	6	7.88^*^	0.19	8	13.17^*^	0.10	4	11.34^*^	0.34
SIS(PS)	10	18.44^*^	0.18	5	11.93^*^	0.47	4	11.7^*^	0.49
**(B) WITH CLINICAL VARIABLES**									
9HPT(A)	10	69.627^*^	0.69	14	71.349^*^	0.68	7	61.391^*^	0.76
9HPT(U)	4	4.187^*^	0.28	4	3.822^*^	0.34	9	1.508^*^	0.9
ARAT(A)	2	5.143^*^	0.96	4	5.723^*^	0.95	2	7.009^*^	0.92
BI	7	9.452^*^	0.43	7	13.378^*^	0.22	12	14.099^*^	0.13
SIS(ADL)	7	16.146^*^	0.78	2	23.484^*^	0.51	11	15.1018	0.8
SIS(HF)	5	9.766^*^	0.03	8	9.4^*^	0.54	5	10.514^*^	0.43
SIS(Mob)	1	16.361^*^	0.37	4	13.523^*^	0.32	6	12.528^*^	0.41
SIS(PS)	4	6.796^*^	0.41	7	4.591^*^	0.64	10	3.817^*^	0.75

*Specific correlates are listed in Table 5*.

(

* *) = significant against chance-level based on permutation-test (p < 0.05); T4 = pre-therapy; T6 = post-therapy; T7 = 1-month post-therapy*.

**Table 5:** List of rs-FC correlates of behavior between all pairs of time-points identified by using linear-kernel SVR are presented below.

**Rank**	**9HPT(A)**	**9HPT(U)**	**ARAT(A)**	**SIS(ADL)**	**SIS(HF)**	**SIS(Mob)**	**SIS(PS)**	**BI**
**(A) WITHOUT CLINICAL VARIABLES**								
**Outcomes at T6 and Input rs-FC Features at T4**								
1	L.SMA-R.M1	L.SMA-R.PMC	R.PMC-L.PMC	R.PMC-R.M1	L.SMA-L.PMC	R.PMC-R.M1	R.SMA-R.M1	L.SMA-R.M1
2	R.SMA-R.M1	R.PMC-R.M1	L.SMA-R.M1	L.Thal-L.M1	R.SMA-R.M1	R.SMA-L.SMA	L.Thal-R.SMA	R.PMC-L.PMC
3	R.M1-L.M1	R.SMA-R.PMC		R.SMA-L.M1	L.SMA-L.M1	L.Thal-R.PMC	R.SMA-L.M1	R.Thal-L.SMA
4	R.PMC-R.M1	L.PMC-R.M1		R.M1-L.M1	R.M1-L.M1	L.Thal-R.M1	L.SMA-L.PMC	
5	R.Thal-L.Thal	L.Thal-L.M1				R.Thal-R.PMC	L.Thal-L.M1	
6	R.Thal-L.M1					R.SMA-R.M1	L.PMC-L.M1	
7	R.SMA-R.PMC						R.M1-L.M1	
8	R.PMC-L.PMC						L.Thal-R.M1	
9	L.Thal-L.M1						R.PMC-L.PMC	
10							R.Thal-L.M1	
**Outcomes at T7 and Input rs-FC Features at T4**								
1	L.SMA-R.M1	L.Thal-L.SMA	R.SMA-R.PMC	R.Thal-L.M1	R.SMA-R.M1	R.PMC-R.M1	R.Thal-R.PMC	L.PMC-L.M1
2	R.SMA-R.M1	R.SMA-L.SMA	L.SMA-R.M1	R.PMC-R.M1	L.PMC-L.M1	L.SMA-L.M1	R.SMA-R.M1	R.SMA-R.M1
3	R.PMC-R.M1	R.Thal-L.PMC	R.SMA-R.M1	L.Thal-R.M1	R.M1-L.M1	R.SMA-L.SMA	R.Thal-L.Thal	R.Thal-L.SMA
4	R.Thal-L.SMA		R.PMC-R.M1	R.SMA-L.M1	L.SMA-L.M1	R.SMA-L.M1	L.Thal-R.M1	L.Thal-L.M1
5	R.M1-L.M1		R.PMC-L.PMC		L.Thal-R.PMC	L.Thal-R.PMC	R.M1-L.M1	R.Thal-L.M1
6	R.SMA-R.PMC		L.PMC-L.M1		R.SMA-L.SMA	L.SMA-L.PMC		
7	L.Thal-R.M1					R.Thal-L.SMA		
8	R.Thal-L.Thal					R.Thal-R.PMC		
9	R.Thal-R.SMA							
10	R.Thal-R.M1							
11	R.PMC-L.PMC							
12	R.Thal-L.M1							
13	L.SMA-R.PMC							
14	L.Thal-L.M1							
**Outcomes at T7 and Input rs-FC Features at T6**								
1	R.PMC-R.M1	L.Thal-R.PMC	R.PMC-R.M1	L.SMA-R.M1	R.PMC-L.M1	R.PMC-R.M1	R.Thal-L.SMA	L.Thal-R.M1
2	R.PMC-L.PMC	L.SMA-R.PMC	R.Thal-R.PMC	R.PMC-L.M1	R.Thal-L.PMC	R.Thal-L.PMC	R.Thal-L.PMC	L.Thal-L.SMA
3	R.Thal-R.PMC	R.SMA-L.M1	L.SMA-R.PMC	R.SMA-R.PMC	L.SMA-R.M1	R.SMA-L.PMC	R.Thal-L.M1	L.SMA-R.PMC
4	R.M1-L.M1	R.Thal-L.M1	R.M1-L.M1	L.SMA-R.PMC	R.Thal-R.M1	R.SMA-L.SMA	R.PMC-L.M1	R.SMA-R.M1
5	L.SMA-R.PMC	R.Thal-R.M1		R.Thal-R.PMC	L.Thal-L.SMA			R.Thal-R.PMC
6	R.SMA-L.PMC	R.PMC-L.M1						L.PMC-L.M1
7								R.SMA-R.PMC
8								R.PMC-R.M1
9								R.M1-L.M1
10								L.Thal-L.M1
11								R.Thal-L.PMC
**(B) WITH CLINICAL VARIABLES**								
**Outcomes at T6 and Input rs-FC and Clinical Features at T4**								
1	Motor Imp.	L.PMC-R.M1	Motor Imp.	TSS	Motor Imp.	L.Thal-R.PMC	NIHSS	Motor Imp.
2	NIHSS	Age	NIHSS	R.SMA-R.PMC	L.SMA-R.PMC		Lesion Hemi	R.Thal-L.SMA
3	Lesion Hemi	L.SMA-L.M1		R.Thal-R.PMC	R.M1-L.M1		R.SMA-R.M1	NIHSS
4	R.M1-L.M1	NIHSS		Motor Imp.	R.PMC-R.M1		L.Thal-R.PMC	Age
5	R.SMA-R.PMC			Lesion Hemi	R.Thal-R.M1			L.SMA-R.M1
6	L.SMA-R.M1			R.Thal-L.M1				R.Thal-L.PMC
7	TSS			R.PMC-R.M1				L.PMC-L.M1
8	R.Thal-L.Thal							
9	R.SMA-R.M1							
10	R.PMC-R.M1							
**Outcomes at T7 and Input rs-FC and Clinical Features at T4**								
1	Motor Imp.	R.SMA-L.SMA	Motor Imp.	R.SMA-L.M1	R.PMC-L.M1	R.PMC-R.M1	Motor Imp.	TSS
2	NIHSS	R.SMA-L.M1	NIHSS	R.Thal-L.M1	Motor Imp.	L.SMA-L.M1	R.Thal-L.Thal	Motor Imp.
3	Lesion Hemi	R.PMC-R.M1	L.Thal-R.PMC		R.SMA-R.M1	R.SMA-L.M1	L.Thal-R.SMA	Age
4	TSS	NIHSS	R.Thal-R.PMC		R.PMC-L.PMC	L.SMA-L.PMC	L.Thal-R.PMC	R.PMC-R.M1
5	R.SMA-R.M1				R.SMA-L.M1		L.Thal-R.M1	R.PMC-L.PMC
6	R.M1-L.M1				L.PMC-L.M1		R.Thal-L.PMC	R.M1-L.M1
7	L.PMC-L.M1				TSS		NIHSS	R.SMA-R.M1
8	L.SMA-R.M1				NIHSS			
9	L.Thal-L.M1							
10	R.Thal-L.SMA							
11	R.Thal-L.PMC							
12	R.Thal-R.SMA							
13	R.SMA-L.M1							
14	R.PMC-R.M1							
**Outcomes at T7 and Input rs-FC and Clinical Features at T6**								
1	Motor Imp.	L.Thal-L.M1	Motor Imp.	R.PMC-L.M1	Motor Imp.	R.Thal-L.PMC	L.SMA-L.PMC	Motor Imp.
2	NIHSS	L.Thal-R.SMA	NIHSS	R.SMA-R.PMC	L.SMA-L.PMC	R.Thal-L.SMA	R.SMA-L.SMA	R.Thal-L.PMC
3	Lesion Hemi	L.Thal-R.PMC		TSS	TSS	R.Thal-L.M1	Motor Imp.	R.PMC-L.PMC
4	R.SMA-L.PMC	R.Thal-L.M1		L.SMA-R.M1	R.Thal-L.PMC	R.PMC-L.PMC	L.Thal-R.M1	R.Thal-R.M1
5	R.PMC-L.PMC	R.SMA-L.M1		NIHSS	NIHSS	L.Thal-L.M1	R.SMA-R.M1	R.Thal-R.PMC
6	L.SMA-R.PMC	NIHSS		L.SMA-L.PMC		R.PMC-L.M1	R.PMC-L.M1	R.Thal-R.SMA
7	R.PMC-R.M1	Gender		R.Thal-R.M1			R.PMC-L.PMC	Age
8		L.Thal-L.SMA		L.PMC-L.M1			NIHSS	TSS
9		R.SMA-R.PMC		R.PMC-R.M1			L.Thal-R.SMA	R.PMC-R.M1
10				R.SMA-L.M1			R.Thal-R.PMC	R.M1-L.M1
11				R.Thal-L.PMC				NIHSS
12								R.SMA-R.M1

*T4 = pre-therapy; T6 = post-therapy; T7 = 1-month post-therapy*.

#### ΔRs-FC as correlates of Δbehavioral outcomes

The performance of SVR using Δrs-FC as correlates of Δbehavioral outcomes are presented in Table 6 (and **ST 2**). SVR models corresponding to ARAT(A) and SIS(HF) performed better than chance-level based on permutation test (*p* < 0.05) as depicted in **SF 4**. Individual predictors involved in estimating the different outcomes are listed in Table 7 (and **ST 5)**. Overall, rs-FC associated with L.M1, R.M1, L.Thal and L.M1, R.M1, R.Thal were the main contributors toward estimation without and with clinical variables respectively. Among the three time-points, better performances were found in cases of correlating Δrs-FC between T6 and T7 and Δbehavioral measures between the same time-period.

**Table 6:** Linear-kernel SVR performances based on leave-one out cross-validation to correlate Δrs-FC between two time-points with Δ behavioral measures between corresponding time-points are presented.

**Outcome**	**Δrs-FC_T6−T4_~Δbehavior_T6−T4_**			**Δrs-FC_T7−T4_~Δbehavior_T7−T4_**			**Δrs-FC_T7−T6_~Δbehavior _T7−T6_**		
	**Features**	**RMSE**	**R^**2**^**	**Features**	**RMSE**	**R^**2**^**	**Features**	**RMSE**	**R^**2**^**
**(A) WITHOUT CLINICAL VARIABLES**									
Δ9HPT(A)	3	110.93	0.22	1	116.69	0.14	4	109.25	0.25
Δ9HPT(U)	1	4.2	0.28	1	4.05	0.26	6	2.86	0.63
ΔARAT(A)	7	20.58^*^	0.33	4	17.87^*^	0.49	6	15.48^*^	0.61
ΔBI	5	8.01	0.18	5	0.05	0.53	6	6.39	0.3
ΔSIS(ADL)	2	10.93	0.24	5	10.62	0.51	8	0.08	0.73
ΔSIS(HF)	5	8.52^*^	0.26	4	26.84^*^	0.36	6	33.47^*^	0.42
ΔSIS(Mob)	4	7.88	0.37	2	13.17	0.1	3	11.34	0.34
ΔSIS(PS)	3	18.44	0.2	3	11.93	0.47	6	0.81	0.2
**(B) WITH CLINICAL VARIABLES**									
Δ9HPT(A)	5	69.63	0.69	3	71.35	0.68	3	61.39	0.76
Δ9HPT(U)	3	4.19	0.28	3	0.1	0.22	3	1.51	0.9
ΔARAT(A)	16	5.14^*^	0.96	8	5.72^*^	0.95	16	7.01^*^	0.92
ΔBI	3	0.05	0.1	7	4.59	0.64	8	3.82	0.75
ΔSIS(ADL)	4	9.45	0.43	5	13.38	0.22	4	14.1	0.13
ΔSIS(HF)	5	16.15^*^	0.78	5	23.48^*^	0.51	4	15.1^*^	0.8
ΔSIS(Mob)	4	9.77	0.03	7	9.4	0.54	5	10.51	0.43
ΔSIS(PS)	4	16.36	0.37	4	13.52	0.32	8	12.53^*^	0.41

Specific correlates are listed in Table 7. (

* *) = significant against chance-level based on permutation-test (p < 0.05); T4 = pre-therapy; T6 = post-therapy; T7 = 1-month post-therapy*.

**Table 7:** List of Δrs-FC correlates of Δbehavior between all pairs of time-points identified by using linear-kernel SVR are presented below.

**Rank**	**Δ9HPT(A)**	**Δ9HPT(U)**	**ΔSIS(ADL)**	**ΔSIS(Mob)**	**ΔSIS(PS)**	**ΔBI**	**ΔARAT(A)**	**ΔSIS(HF)**
**(A) WITHOUT CLINICAL VARIABLES**								
**Outcomes at T6 and Input Δrs-FC Features at T4**								
1	R.Thal-R.SMA	L.SMA-L.PMC	L.SMA-R.PMC	R.PMC-R.M1	L.SMA-R.M1	R.Thal-L.PMC	L.Thal-L.M1	R.PMC-L.M1
2	R.Thal-R.M1		L.Thal-R.PMC	L.Thal-L.M1	R.SMA-L.SMA	L.SMA-L.PMC	L.Thal-L.PMC	R.SMA-R.M1
3	R.SMA-R.PMC			R.PMC-L.M1	R.PMC-L.M1		L.SMA-L.PMC	L.PMC-L.M1
4				L.Thal-R.PMC			L.SMA-R.M1	L.Thal-R.SMA
5							L.SMA-R.PMC	L.PMC-R.M1
6							R.M1-L.M1	
7							L.Thal-R.PMC	
**Outcomes at T7 and Input Δrs-FC Features at T4**								
1	R.Thal-L.SMA	L.Thal-R.PMC	R.Thal-R.M1	L.Thal-L.M1	R.Thal-L.PMC	L.Thal-R.PMC	R.Thal-R.SMA	R.Thal-L.M1
2			L.Thal-R.PMC	R.Thal-R.M1	R.SMA-L.M1	L.PMC-L.M1	L.Thal-L.PMC	L.PMC-L.M1
3			R.PMC-R.M1		R.SMA-R.M1	R.SMA-R.PMC	L.Thal-L.M1	R.Thal-R.PMC
4			R.PMC-L.PMC			R.SMA-R.M1	R.Thal-L.SMA	L.SMA-L.M1
5			L.SMA-L.M1			L.SMA-R.M1		
**Outcomes at T7 and Input Δrs-FC Features at T6**								
1	L.SMA-L.PMC	R.PMC-R.M1	R.Thal-R.SMA	R.SMA-R.M1	L.Thal-R.SMA	R.SMA-L.SMA	L.Thal-L.M1	L.SMA-L.M1
2	L.SMA-R.PMC	R.PMC-L.PMC	L.Thal-R.SMA	R.Thal-R.M1	L.Thal-R.M1	L.PMC-R.M1	L.Thal-L.SMA	L.Thal-L.M1
3	R.M1-L.M1	L.SMA-R.PMC	R.SMA-R.PMC	R.PMC-L.M1	R.Thal-R.PMC	R.SMA-R.M1	R.Thal-L.PMC	L.SMA-L.PMC
4	R.SMA-L.M1	R.SMA-L.PMC	R.SMA-R.M1		L.SMA-L.M1	R.Thal-L.PMC	L.Thal-R.PMC	L.Thal-R.PMC
5		L.Thal-L.M1	R.PMC-L.PMC		R.PMC-L.M1	R.SMA-R.PMC	R.Thal-R.SMA	L.Thal-L.SMA
6		L.Thal-L.SMA	L.SMA-L.PMC		L.Thal-L.M1	L.PMC-L.M1	R.PMC-R.M1	L.PMC-L.M1
7			L.Thal-L.PMC					
8			L.PMC-R.M1					
**(B) WITH CLINICAL VARIABLES**								
**Outcome at T6 and Input Δrs-FC at T4 + Clinical Features**								
1	R.Thal-R.SMA	NIHSS	L.SMA-R.PMC	R.Thal-L.M1	L.SMA-R.M1	R.PMC-L.M1	NIHSS	R.PMC-L.M1
2	Gender	R.Thal-L.PMC	L.Thal-R.PMC	L.Thal-R.M1	L.SMA-L.M1	R.Thal-L.PMC	Motor Imp.	R.Thal-L.SMA
3	R.SMA-L.M1	L.SMA-R.PMC	TSS	R.PMC-R.M1	R.SMA-L.SMA	R.SMA-L.M1	L.SMA-R.PMC	R.Thal-R.SMA
4	R.Thal-R.M1		R.M1-L.M1	L.SMA-R.M1	L.Thal-R.M1		R.Thal-L.PMC	L.Thal-R.M1
5	R.Thal-L.PMC						Lesion Hemi	R.SMA-L.PMC
6							R.Thal-L.SMA	
7							L.SMA-L.PMC	
8							L.SMA-L.M1	
9							R.Thal-R.SMA	
10							R.PMC-R.M1	
11							L.SMA-R.M1	
12							R.M1-L.M1	
13							R.SMA-R.M1	
14							L.Thal-L.M1	
15							R.PMC-L.M1	
16							TSS	
**Outcome at T7 and Input Δrs-FC at T4 + Clinical Features**								
1	R.Thal-L.SMA	R.SMA-R.PMC	Motor Imp.	R.Thal-R.M1	R.SMA-L.M1	L.PMC-L.M1	L.Thal-L.PMC	R.Thal-L.M1
2	NIHSS	R.Thal-R.M1	R.Thal-R.M1	Age	R.Thal-L.PMC	L.Thal-R.PMC	NIHSS	L.PMC-L.M1
3	R.Thal-R.PMC	Lesion Hemi	L.SMA-R.PMC	L.Thal-L.M1	R.PMC-L.M1	R.SMA-R.M1	R.SMA-L.M1	R.Thal-R.PMC
4			R.SMA-L.M1	R.Thal-L.SMA	Lesion Hemi	L.Thal-L.M1	R.Thal-R.SMA	L.Thal-R.PMC
5			R.SMA-R.PMC	L.Thal-L.PMC		R.M1-L.M1	R.Thal-L.M1	R.SMA-L.M1
6				Motor Imp.		R.Thal-L.PMC	L.Thal-R.PMC	
7				L.Thal-R.SMA		NIHSS	L.PMC-R.M1	
8							L.SMA-L.PMC	
**Outcome at T7 and Input Δrs-FC at T6 + Clinical Features**								
1	R.Thal-R.SMA	R.PMC-R.M1	L.SMA-L.PMC	R.SMA-R.M1	L.SMA-R.M1	R.SMA-L.SMA	L.Thal-L.SMA	L.SMA-L.M1
2	L.PMC-L.M1	L.SMA-R.PMC	R.SMA-R.M1	R.Thal-R.M1	Lesion Hemi	L.PMC-R.M1	L.Thal-L.PMC	L.Thal-L.M1
3	R.PMC-L.PMC	R.SMA-L.PMC	L.SMA-R.M1	Motor Imp.	R.SMA-L.PMC	L.Thal-L.PMC	L.Thal-L.M1	R.SMA-R.PMC
4			L.Thal-L.PMC	R.Thal-L.Thal	R.Thal-L.Thal	R.Thal-L.PMC	R.SMA-R.PMC	R.Thal-L.Thal
5				R.PMC-L.M1	L.Thal-R.PMC	R.SMA-L.M1	Age	
6					L.SMA-L.M1	R.Thal-L.SMA	TSS	
7					L.PMC-L.M1	R.Thal-R.PMC	L.PMC-R.M1	
8					NIHSS	L.Thal-R.M1	L.SMA-L.M1	
9							R.Thal-L.Thal	
10							R.M1-L.M1	
11							L.PMC-L.M1	
12							Gender	
13							L.Thal-R.SMA	
14							NIHSS	
15							L.Thal-R.PMC	
16							L.Thal-R.M1	

#### Behavioral correlates at preceding stages of behavioral outcomes at succeeding stages

The performance of SVR using behavioral measures at preceding time-points as correlates of behavioral outcomes at succeeding time-points are presented in Table 8 (and **ST 3**). All the SVR models performed better than chance-level based on permutation test (*p* < 0.05) as depicted in **SF 5**. Individual predictors involved in estimating the different outcomes are listed in Table 9 (and **ST 6)**. Overall, the behavioral measures from the preceding time-point were almost always the highest-ranked correlates, relative to the clinical variables. Among the three time-points, better overall performances were found in cases of correlating behavior at T4 with those at T6.

**Table 8:** Linear-kernel SVR performances based on leave-one out cross-validation to correlate behavioral measures at preceding time-point and clinical variables with behavioral measures at succeeding time-point are presented.

**Outcome**	**T4 behavior~T6 behavior**			**T4 behavior~T7 behavior**			**T6 behavior~T7 behavior**		
	**Features**	**RMSE**	**R^**2**^**	**Features**	**RMSE**	**R^**2**^**	**Features**	**RMSE**	**R^**2**^**
9HPT(A)	2	2.52^*^	0.74	1	3.28^*^	0.52	3	3.25^*^	0.53
9HPT(U)	4	37.36^*^	0.91	5	23.4^*^	0.97	5	9.05^*^	0.99
ARAT(A)	3	2.73^*^	0.99	3	3.31^*^	0.98	3	3.13^*^	0.98
BI	1	5.48^*^	0.62	2	5.19^*^	0.54	3	4.83^*^	0.6
SIS(ADL)	3	8.56^*^	0.54	3	10.24^*^	0.54	3	11.42^*^	0.43
SIS(HF)	4	12.74^*^	0.86	4	13.03^*^	0.85	4	7.9^*^	0.94
SIS(Mob)	4	5.36^*^	0.71	2	11.8^*^	0.28	3	10.09^*^	0.47
SIS(PS)	2	14.7^*^	0.49	2	12.03^*^	0.46	3	8.85^*^	0.71

Specific correlates are listed in Table 9. (

* *) = significant against chance-level based on permutation-test (p < 0.05); T4 = pre-therapy; T6 = post-therapy; T7 = 1-month post-therapy*.

**Table 9:** List of behavioral and clinical correlates at preceding time-points using linear-kernel SVR for estimation of measures at succeeding time-points are presented below.

**Rank**	**9HPT(A)**	**9HPT(U)**	**ARAT(A)**	**SIS(ADL)**	**SIS(HF)**	**SIS(Mob)**	**SIS(PS)**	**BI**
**Outcome at T6 and Input Behavior at T4 + Clinical Variables**								
1	9HPT(A)	9HPT(U)	Motor Imp.	SIS(ADL)	SIS(HF)	SIS(Mob)	SIS(PS)	BI
2	NIHSS	Motor Imp.	ARAT(A)	Lesion Hemi	NIHSS	NIHSS	NIHSS	
3		NIHSS	NIHSS	NIHSS	TSS	TSS		
4		Lesion Hemi			Motor Imp.	Lesion Hemi		
**Outcome at T7 and Input Behavior at T4 + Clinical Variables**								
1	9HPT(A)	9HPT(U)	ARAT(A)	SIS(ADL)	SIS(HF)	SIS(Mob)	SIS(PS)	BI
2		NIHSS	Motor Imp.	Motor Imp.	NIHSS	Motor Imp.	Motor Imp.	TSS
3		Motor Imp.	NIHSS	TSS	TSS			
4		Lesion Hemi			Motor Imp.			
5		TSS						
**Outcomes at T7 and Input Behavior at T6 + Clinical Variables**								
1	9HPT(A)	9HPT(U)	ARAT(A)	SIS(ADL)	SIS(HF)	SIS(Mob)	SIS(PS)	BI
2	TSS	Lesion Hemi	Motor Imp.	Motor Imp.	Motor Imp.	Motor Imp.	Age	TSS
3	Motor Imp	Motor Imp.	NIHSS	TSS	NIHSS	NIHSS	Motor Imp.	Motor Imp.
4		TSS			TSS			
5		NIHSS						

*T4 = pre-therapy; T6 = post-therapy; T7 = 1-month post-therapy*.

### Impact of clinical variables

We tested each SVR model with and without the impact of the identified clinical variables to account for potential confounding effects they might have. In general, the SVR performance improved upon addition of clinical variables as input features. Contribution of individual clinical variables, relative to rs-FC, Δrs-FC and behavioral input features can be found in **ST 4-6** respectively. The most involved clinical features were: NIHSS, motor impairment severity for ANALYSIS I and III and NIHSS, motor impairment severity and lesion hemisphere for ANALYSIS II. In terms of ROI contribution, rs-FC associated with L.M1, R.M1 and R.PMC were the important contributors for ANALYSIS I even after adjusting for clinical confounds. For ANALYSIS II, the important contributors included L.M1, R.M1 and L.Thal without clinical variables and L.M1, R.M1, and R.Thal with clinical variables.

### Linear vs. non-linear regression

The overall performances of the linear and non-linear SVR models were compared in terms of their RMSE values computed via LOOCV (**SF 6–8**). Comparing the RMSE values revealed that the linear and non-linear SVR models performed approximately similarly with the non-linear model being slightly more generalizable with lower error when rs-FC and Δrs-FC were used as input variables. When behavioral measures were used as input variables, linear SVR appeared to perform better.

## Discussion

### Impact of bci intervention based on identified correlates

The objective of this study was to assess behavioral outcomes following the described BCI intervention. To do so, rs-FC, Δrs-FC, and behavioral measures were utilized. Evaluation of outcomes at the third time-point, namely the 1-month post-intervention, would be particularly important to understand the potential long-term impact of the intervention. As would be expected, behavioral measures at preceding time-points estimated the behavioral measures at succeeding time-points better than rs-FC or Δrs-FC. However, using behavioral measures alone does not provide the knowledge of possible neural reorganization in the brain. Neuroimaging-based rs-FC features can offer this complementary information and serve as an alternative means to assess outcomes. In comparison to pre-intervention measures, the post-intervention input (rs-FC, Δrs-FC, behavioral) measures were more indicative of outcomes at 1-month post-intervention. That could suggest neural reorganization occurring between pre- and post-intervention that is at least partially retained at 1-month post-therapy.

### Rs-fc as a tool for predicting behavioral changes

FMRI has been shown as a useful biomarker in predicting the impact of several forms of rehabilitation on the recovery of function in the stroke population (Johansen-Berg et al., 2002; Ward et al., 2003a; Sharma et al., 2009; Várkuti et al., 2013; Young et al., 2014b). Rs-fMRI, in particular, is a useful non-invasive method used to study impaired subjects such as stroke survivors, as it is time-efficient and task-free, reducing the burden on study participants. In our study, the impact of BCI intervention was examined using rs-FC and associated changes corresponding to several objective and subjective behavioral outcomes. Rs-FC as correlates formed reliable SVR models across all outcomes. However, with Δrs-FC, models corresponding to ARAT(A) and SIS(HF) were only significant above chance-level. ARAT(A) and SIS(HF) are objective and subjective measures of impairment due to stroke and ability to use the impaired hand respectively. Improvement in these outcomes following the intervention demonstrates the impact of BCI-aided therapy. The models that were not significant against chance level could potentially be due to low variability in the normalized outcomes as well as limited sample size. Additionally, the main contributing regions remained focused on bilateral M1 areas with and without the influence of the clinical features. These findings illustrate that rs-FC serves as a stable imaging biomarker in understanding the functional correlates of the recovery process and could, thus, guide future rehabilitative studies in tracking changes over time.

### Machine learning as a tool for predictive modeling

In the context of fMRI studies, fewer studies have used prediction of outcomes on a continuous scale (Ganesh et al., 2008; Dosenbach et al., 2010; Michel et al., 2011; Vergun et al., 2013), where SVR-based models have been adopted to address different parts of data analysis, the majority of which, are based on a simple linear-kernel SVR. Even fewer studies have explored the improved performance offered by non-linear kernels. For instance, non-linear SVR has been incorporated in the preprocessing pipeline of fMRI data to accurately detect activation by accounting for intrinsic spatio-temporal autocorrelations (Wang et al., 2003) and cognitive states of participants in a virtual reality environment have been predicted based on fMRI data using non-linear SVR (Di Bono and Zorzi, 2008). With inclusion of non-linear-kernel SVR, our work adds to the growing literature that provides insight on adopting the more generalizable non-linear approaches for regression based on fMRI data. This could indicate that while the underlying relationship between rs-FC and behavioral measures might not necessarily be linear, the relationship within a given behavioral measure could be better expressed linearly. While linear models were useful in interpreting the contributing features, non-linear models performed slightly better in explaining possible non-linear interactions with better generalizability. Our findings suggest promise in that, given fMRI data from a large cohort, machine learning-based regression models may be trained to predict behavioral change resulting from BCI intervention on a single-subject level. From the clinical perspective, such an application could serve as a supplementary prognostic tool for patients and their families in estimating the timeline and/or capacity of potential recovery through this intervention.

### The bigger picture

Our work adds to the ongoing investigation of understanding the trajectory of motor recovery in the chronic stage of stroke as a result of BCI-aided rehabilitative intervention using a data-driven approach. These findings are in line with works that suggest that using rehabilitative therapies have enabled recovery even at the chronic stage of stroke (Fasoli et al., 2003; Caria et al., 2011). This means that even though motor recovery associated with the paretic side might have plateaued, there could still be potential for further recovery. This was evident from the predominant involvement of rs-FC and Δrs-FC associated with the bilateral M1, which is primarily known to be a center for voluntary motor behavior including but not limited to movement planning, movement initiation and motor learning. While the roles of neuroimaging methods such as task-fMRI (Young et al., 2014b) and diffusion images (Song et al., 2014) in relation to motor recovery facilitated by BCI in our cohort have been explored, the current study fills a gap by examining rs-fMRI as a potential biomarker for recovery. Since it is established that activations identified by task-fMRI have overlapping functional areas with rs-fMRI within the motor network (Biswal et al., 1995), it allows us to draw parallels between our study and those based on task-fMRI. Additionally, thalamic Δrs-FC also emerged as a region with strong involvement in estimating changes in ARAT(A) and SIS(HF), which was demonstrated using task-fMRI activation associated with the same outcomes in our precedent study (Young et al., 2014b). Another task-fMRI-based study by Ward et al. (2003b) also reported thalamic correlations with motor recovery especially in stroke subjects (time since stroke onset > 3 months) with MCA lesions. It could be possible that our findings are similar as half of the subjects included in our study exhibited MCA lesions as well. From data modeling perspective, while traditional methods such as general linear models assume a certain distribution of data, SVR offers a non-parametric method that can model both linear and non-linear relationships in the data and adds to the growing body of studies using machine learning prediction models to analyze fMRI (Di Bono and Zorzi, 2008; Dosenbach et al., 2010; Vergun et al., 2013).

### Limitations

This study highlights how machine learning holds potential to provide useful information by correlating neuroimaging changes to behavioral changes. However, the results can be limited by the sample size that can, in turn, affect the capability of drawing generalizable conclusions as machine learning models such as SVR are typically based on training on data from a much larger cohort. Involvement of NIHSS stroke severity as a feature across multiple outcomes could suggest that lesion size and/or volume might be an important consideration (Chen et al., 2000; Shelton and Reding, 2001) and should be included in future analysis. Feature selection, realized by SFS, was important in deciding the role of relevant correlates of each behavioral scale. However, SFS suffers from the drawback that it cannot remove features from the model that become obsolete upon addition of new features. Recent work suggested that rs-FC can be quantified in several ways using metrics such as cosine similarity and dynamic time warping (Smith et al., 2011). Thus, the choice of metric used for rs-FC might affect the features selected for each outcome.

### Future scope

With ongoing recruitment, a larger and more generalizable prediction model could be developed by considering the following. The complete BCI-aided intervention involved both imaging as well as behavioral data at multiple distinct time points, of which only pre-, post- and 1-month post-intervention data have been used in the current analysis. With a larger sample size, the analysis, therefore, could be expanded further by considering the changes in rs-FC over other time-points and correlating them with corresponding behavioral outcomes and changes. Since recovery is a multi-faceted process, other imaging methods, such as diffusion tensor images, structural images, and perfusion images can provide complementary information about brain changes and could be incorporated as features to SVR. Potentially, multiple of these neuroimaging methods could be combined so as to assess the relative importance of each as a biomarker of stroke recovery through the BCI-intervention. Correlation and interaction among the different behavioral measures could be simultaneously accounted for by implementing a multiple-output SVR that uses a single model to predict multiple outcomes. Additionally, differences and similarities among predictors between stroke subjects and matched healthy subjects undergoing the BCI-intervention will help to further understand the impact of this intervention.

## Conclusion

We showed that rs-FC, changes in rs-FC and early-stage behavior can estimate behavioral outcomes and changes in chronic-stage stroke subjects following this BCI-aided intervention for rehabilitation. Machine learning-based SVR models helped to identify specific correlates of for objective as well as subjective behavioral scales. Among the neural substrates identified, important regions contributing to the estimation involved the left and right primary motor areas. Linear and non-linear kernels for SVR indicated similar results with non-linear SVR being slightly more accurate in estimating the outcomes and forming more generalizable models. The results, however, were more interpretable using the linear-kernel models. For further research, the kernel for SVR must be chosen based on the trade-off between lower error rates and interpretability. Given the promise of this kind of BCI intervention in stroke rehabilitation, the coupling of machine learning with neuroimaging and behavioral measures can aid further identification of neuroplastic changes corresponding to behavioral outcomes to estimate and track stroke recovery, both in terms of neural reorganization and improvements to motor function.

## Data availability statement

The raw data supporting the conclusions of this manuscript will be made available by the authors, without undue reservation, to any qualified researcher.

## Author contributions

RM was involved in data collection, analysis, interpretation of results and writing of the manuscript. AS was involved in data collection, preprocessing data and writing the manuscript. BY was involved in subject recruitment, data collection and editing of the manuscript. AR, KD, TJ, MM, JT, and HA were involved in data collection. VN contributed to data collection, manuscript editing and intellectual content. TK was involved in the recruitment of study participants. KC was involved in subject recruitment. DE is the co-I and JW, VP are co-PIs and were involved in study conception, design, manuscript editing, intellectual content and supervised all aspects of the study.

### Conflict of interest statement

The authors declare that the research was conducted in the absence of any commercial or financial relationships that could be construed as a potential conflict of interest.

## Abbreviations

9HPT: Nine Hole Peg Test
ARAT: Action Research Arm Test
BCI: brain-computer interface
BI: Barthel Index
BOLD: blood-oxygen-level dependent
EEG: Electroencephalogram
FES: functional electrical stimulation
fMRI: functional magnetic resonance imaging
LOOCV: leave-one-out cross validation
M1: primary motor area
MCA: middle cerebral artery
MNI: Montreal Neurological Institute
MSE: mean squared error
NIHSS: National Institute of Health Stroke Scale
PMC: premotor cortex
RMSE: root mean squared error
rs-FC: resting state functional connectivity
Δrs-FC: change in resting-state functional connectivity
SIS: Stroke Impact Scale
SMA: supplementary motor area
SVM: support vector regression
SVR: support vector regression
T1-T3: control period (no intervention)
T4: pre-intervention
T6: post-intervention
T7: one-month post-intervention
TS: tongue stimulation
SF: Supplementary Figure
ST: Supplementary Table.
