# Supplementary material for: Early Findings on Functional Connectivity Correlates of Behavioral Outcomes of Brain-Computer Interface Stroke Rehabilitation Using Machine Learning
Source: Front Neurosci. 2018 Sep 11;12:624. doi: 10.3389/fnins.2018.00624 (PMC6142044; doi:10.3389/fnins.2018.00624)

Supplementary Material

Early Findings on Functional Connectivity Correlates of Behavioral Outcomes of Brain-Computer Interface Stroke Rehabilitation using Machine Learning

Rosaleena Mohanty*****, Anita M. Sinha, Alexander Remsik, Keith Dodd, Tyler Jacobson, Matthew McMillan, Jaclyn Thoma, Hemali Advani, Brittany M. Young, Veena A. Nair, Theresa J. Kang, Kristin Caldera, Dorothy F. Edwards, Justin C. Williams, Vivek Prabhakaran

*** Correspondence:** Rosaleena Mohanty: rmohanty@wisc.edu

1. Principle behind support vector regression

Consider pairs of N data points, $\{(x_1, y_1), \dots (x_N, y_N)\} \subset X \times \mathbb{R}$, X being the input space and $\mathbb{R}$ representing real numbers. The x_i represents the input features, and the y_i represents the outcome variable. Here, x_i encapsulates the input features (rs-FC, Δrs-FC and behavioral measures at preceding time-points) corresponding to each subject, and y_i stands for the outcomes (behavioral measures and changes in them) such as 9HPT, ARAT, BI, and SIS measures. In order to avoid dominance of one feature over others, each feature x_i is standardized to x_i^* so as to be comparable. We chose to standardize features this way in place of the traditional Fisher z-transformation of rs-FC for two reasons: (a) SVR does not assume a normal distribution of data and (b) while Fisher z-transformation, typically applied to correlation coefficients, may be applicable to the rs-FC data, it might not be a suitable standardization for additional confounding variables such as age, gender, etc. Standardization is essential, especially when the features are not measured in the same units, to make the involved features insensitive to the scales on which they are measured. Standardization was performed as follows:

$$\boldsymbol{x_i^*} = \frac{\boldsymbol{x_i} - \boldsymbol{\mu^*}}{\boldsymbol{\sigma^*}} \tag{1}$$

where, $\mu^* = \frac{1}{N}\sum_{k=1}^{N} x_k$ and $(\sigma^*)^2 = \frac{N}{N^2 - N}\sum_k (x_k - \mu^*)^2$

The goal of SVR is to find a linear or non-linear function that fits the data x_i^* such that the estimated value of y_i lies within a specified small margin of error ε from the true value of y_i. If $f(\boldsymbol{x}^*)$ is the estimated function, we have:

$$\boldsymbol{f}(\boldsymbol{x}^*) = < \boldsymbol{w}, \boldsymbol{\Phi}(\boldsymbol{x}^*) > + \boldsymbol{b} \tag{2}$$

where $w \in X$ is the weight vector, $\Phi(\boldsymbol{x}^*)$ is the mapping function that can be linear or non-linear, $b \in \mathbb{R}$ is the bias term, and $<,>$ denotes the dot product in X.

When the weights w turn out to be small, the estimated y_i will deviate from true y_i within a small margin. This problem can be rewritten in the form of a convex optimization problem as follows:

$$\boldsymbol{minimize}\ \frac{1}{2}||\boldsymbol{w}||^2 \tag{3}$$

$$\boldsymbol{subject\ to} \begin{cases} \boldsymbol{y_i} - < \boldsymbol{w}, \boldsymbol{\Phi}(x_i^*) > -\boldsymbol{b} \leq \boldsymbol{\varepsilon} \\ < \boldsymbol{w}, \boldsymbol{\Phi}({}_{\boldsymbol{i}}x_i^*) > +\boldsymbol{b} - \boldsymbol{y_i} \leq \boldsymbol{\varepsilon} \end{cases} \tag{4}$$

where, ε is the small allowable margin of error between true y_i and estimated y_i.

The above can be reformulated again to cope with possible infeasible constraints that may arise in the optimization problem by incorporating slack variables. This is analogous to the concept of "soft margin" in SVM classifiers that allow for marginally higher error to get a superior performance overall. The reformulation, then, becomes:

$$\boldsymbol{minimize}\ \frac{1}{2}||\boldsymbol{w}||^2 + \boldsymbol{C}\sum_{\boldsymbol{i=1}}^{\boldsymbol{l}}(\boldsymbol{\zeta_i} + \boldsymbol{\zeta_i^*}) \tag{5}$$

$$\boldsymbol{subject\ to} \begin{cases} \boldsymbol{y_i} - < \boldsymbol{w}, \boldsymbol{\Phi}(\ {}_{\boldsymbol{i}}x_i^*) > -\boldsymbol{b} \leq \boldsymbol{\varepsilon} + \boldsymbol{\zeta_i} \\ < \boldsymbol{w}, \boldsymbol{\Phi}(\ x_i^*) > +\boldsymbol{b} - \boldsymbol{y_i} \leq \boldsymbol{\varepsilon} + \boldsymbol{\zeta_i^*} \\ \boldsymbol{\zeta_i}, \boldsymbol{\zeta_i^*} \geq \boldsymbol{0} \end{cases} \tag{6}$$

where $C > 0$ is a constant that determines the extent to which error more than ε is allowed. The given method for regression can be applied to the linear case where a linear estimate between input and output values is made. With the use of SVM, a kernel method [54] can be applied to estimate non-linear relationships between the input and the output variables as well, giving a better fit to the data. We implemented the SVR using a linear as well as a Gaussian radial basis function as kernels. The kernels for linear and non-linear cases are given by (7) and (8) respectively:

$$\boldsymbol{k}(x_i^*, x_j^*) = x_i^{*\boldsymbol{T}} x_j^*{}_{\boldsymbol{i}}\ \ \boldsymbol{x_j} \tag{7}$$

$$k(x_i^*, x_j^*) = \exp\left(-\frac{||x_i^* - x_j^* \quad ||^2}{2\sigma^2}\right) \quad (8)$$

where ${x_i}^{*T} x_j^*$ represents the dot product between two input feature points, $||x_i^* - x_j^*||^2$ is the squared Euclidean distance between two input feature points, and σ is a free parameter. The kernel maps the input feature dataset onto a higher-dimensional feature space where a linear separation may exist.

In the case of linear regression, $f(\boldsymbol{x}^*)$ is determined in the input space, so it is possible to derive the weights w as a linear combination of the training patterns from the optimality constraints based on the dual problem. However, in the case of non-linear regression, weights w cannot be derived explicitly since the function $f(\boldsymbol{x}^*)$ is no longer found in the input space but in the feature space using the kernel function.

SF 1. Comparison of group medians at three time-points (N=20): pre-intervention (T4), post-intervention (T6) and one-month post-intervention (T7). SIS (Mob), SIS (HF), ARAT (A) showed higher values at T7 relative to T4 and T6 (p > 0.05; Mann Whitney U-test).

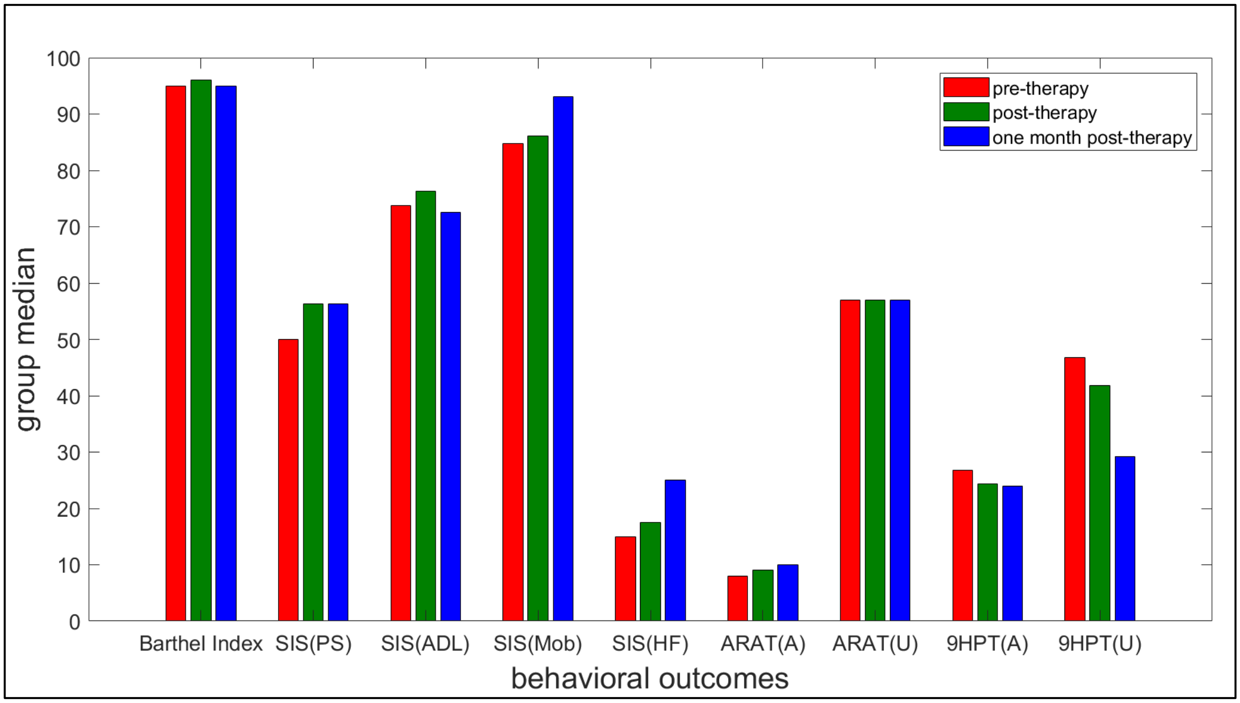

SF 2. Comparison of group medians during the control phase (N=10): T1, T2, T3 and T4 (pre-intervention). Mann Whitney U-test was used to verify that there were no significant differences across each pair of time-points for each measure (p>0.05).

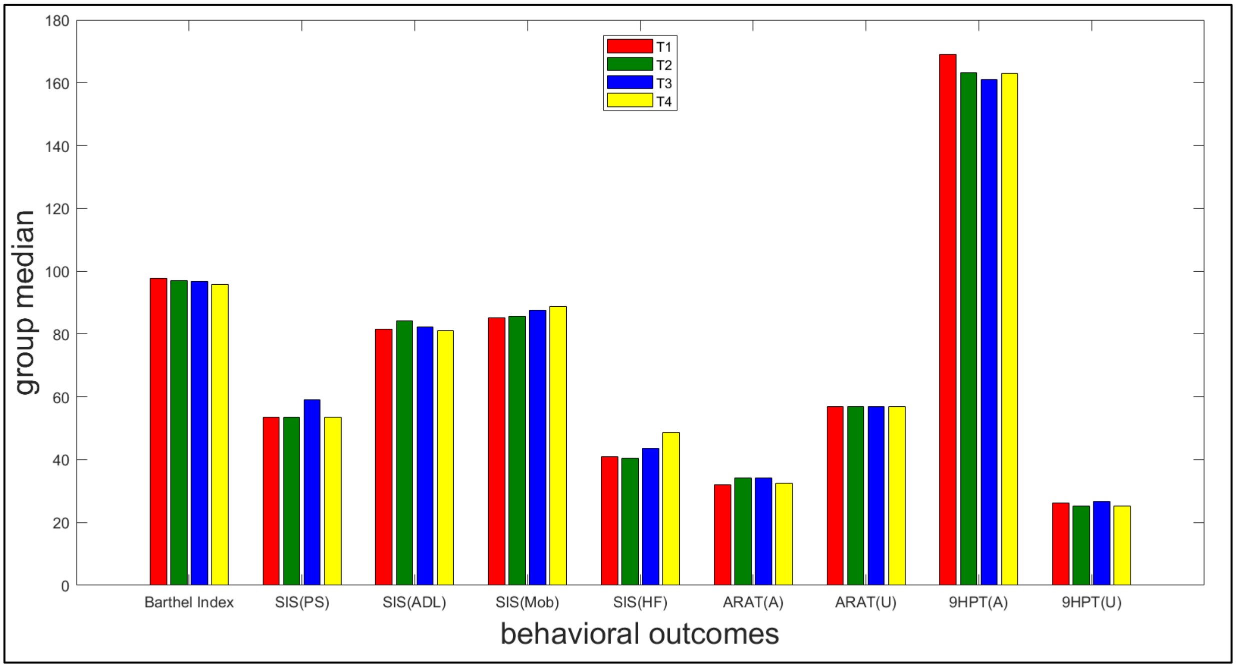

ST 1. Non-linear SVR performances based on LOOCV to correlate rs-FC at preceding time-point with behavioral measures at succeeding time-point are presented. Specific correlates are listed in **ST 4**. (*****) = significant against chance-level based on permutation-test (p<0.05); T4 = pre-intervention; T6 = post-intervention; T7 = one-month post-intervention.

**(A) Without Clinical Variables**						
**Outcome**	**T4 rs-FC ~ T6 behavior**		**T4 rs-FC ~ T7 behavior**		**T6 rs-FC ~ T7 behavior**	
	**Features**	**RMSE**	**Features**	**RMSE**	**Features**	**RMSE**
**9HPT(A)**	2	77.8*	2	127.51*	2	68.82*
**9HPT(U)**	4	3.89*	3	4.07*	2	3.548*
**ARAT(A)**	3	14.77*	3	16.51*	3	17.71*
**BI**	2	7.38*	5	7.83*	3	5.32*
**SIS(ADL)**	2	12.6*	2	15.958*	3	12.64*
**SIS(HF)**	3	32.14*	2	35.26*	2	30.53*
**SIS(Mob)**	2	9.94*	2	7.768*	3	14.68*
**SIS(PS)**	3	20.58*	3	13.95*	4	11.27*

(B) With Clinical Variables						
**Outcome**	**T4 rs-FC ~ T6 behavior**		**T4 rs-FC ~ T7 behavior**		**T6 rs-FC ~ T7 behavior**	
	**Features**	**RMSE**	**Features**	**RMSE**	**Features**	**RMSE**
**9HPT(A)**	3	22.99*	3	28.41*	1	64.04*
**9HPT(U)**	4	3.44*	2	2.65*	1	4.76*
**ARAT(A)**	3	4.41*	3	5.54*	1	5.45*
**BI**	4	12.6*	5	11.71*	4	15.33*
**SIS(ADL)**	5	10.14*	3	10.22*	3	13.25*
**SIS(HF)**	3	7.88*	2	14.68*	3	9.7*
**SIS(Mob)**	3	12.55*	1	14.43*	4	7.83*
**SIS(PS)**	3	5.91*	2	5.02*	3	5.01*

ST 2. Non-linear SVR performances based on LOOCV to correlate Δrs-FC between two time-points with Δbehavioral measures between corresponding time-points are presented. Specific correlates are listed in **ST 5**. (*****) = significant against chance-level based on permutation-test (p<0.05); T4 = pre-intervention; T6 = post-intervention; T7 = one-month post-intervention.

(A) Without Clinical Variables						
**Outcome**	**Δrs-FC$_{T6-T4}$ ~ Δbehavior $_{T6-T4}$**		**Δrs-FC$_{T7-T4}$ ~ Δbehavior $_{T7-T4}$**		**Δrs-FC$_{T7-T6}$ ~ Δbehavior $_{T7-T6}$**	
	**Features**	**RMSE**	**Features**	**RMSE**	**Features**	**RMSE**
**Δ9HPT(A)**	5	77.8	8	127.51	3	68.82
**Δ9HPT(U)**	3	3.89	4	4.07	3	3.54
**ΔARAT(A)**	4	14.77*	2	16.51*	3	17.71*
**ΔBI**	2	7.38	3	7.83	2	5.32
**ΔSIS(ADL)**	3	12.6	11	15.95	6	12.64
**ΔSIS(HF)**	4	32.14*	6	35.26*	1	30.53*
**ΔSIS(Mob)**	3	9.94	7	7.76	3	14.68
**ΔSIS(PS)**	6	20.58	2	13.95	4	11.27

(B) With Clinical Variables						
**Outcome**	**Δrs-FC$_{T6-T4}$ ~ Δbehavior $_{T6-T4}$**		**Δrs-FC$_{T7-T4}$ ~ Δbehavior $_{T7-T4}$**		**Δrs-FC$_{T7-T6}$ ~ Δbehavior $_{T7-T6}$**	
	**Features**	**RMSE**	**Features**	**RMSE**	**Features**	**RMSE**
**Δ9HPT(A)**	3	22.99	3	28.41	4	64.04
**Δ9HPT(U)**	3	3.44	4	2.65	2	4.76
**ΔARAT(A)**	3	4.41*	3	5.54*	7	5.45*
**ΔBI**	3	5.91	5	5.02	2	5.01
**ΔSIS(ADL)**	3	12.6	4	11.71	4	15.33
**ΔSIS(HF)**	2	10.14*	6	10.22*	1	13.25*
**ΔSIS(Mob)**	3	7.88	5	14.68	3	9.7
**ΔSIS(PS)**	6	12.55	6	14.43	6	7.83

ST 3. Non-linear SVR performances based on LOOCV to correlate behavioral measures at preceding time-point and clinical variables with behavioral measures at succeeding time-point are presented. Specific correlates are listed in **ST 6**. (*****) = significant against chance-level based on permutation-test (p<0.05); T4 = pre-intervention; T6 = post-intervention; T7 = one-month post-intervention.

**Outcome**	**T4 behavior ~ T6 behavior**		**T4 behavior ~ T7 behavior**		**T6 behavior ~ T7 behavior**	
	**Features**	**RMSE**	**Features**	**RMSE**	**Features**	**RMSE**
**9HPT(A)**	2	2.18*	1	4.72*	2	3.09*
**9HPT(U)**	2	125.18*	1	30.63*	1	132.76*
**ARAT(A)**	3	2.2*	3	26.32*	2	2.99*
**BI**	1	4.45*	3	8.04*	3	5.84*
**SIS(ADL)**	2	11.47*	3	15.95*	1	10.34*
**SIS(HF)**	2	10.49*	2	12.288*	1	35.37
**SIS(Mob)**	2	6.61*	2	14.53*	2	10.28*
**SIS(PS)**	2	15.76*	1	13*	2	10.57*

ST 4. List of rs-FC correlates of behavior between all pairs of time-points from non-linear SVR are presented below.

**(A) Without Clinical Variables**								
**9HPT(A)**	**9HPT(U)**	**ARAT(A)**	**SIS(ADL)**	**SIS(HF)**	**SIS(Mob)**	**SIS(PS)**	**BI**	**Outcomes at T6 Input rs-FC Features at T4**
L.SMA-R.M1	R.M1-L.M1	R.PMC-L.M1	R.SMA-R.PMC	R.M1-L.M1	L.PMC-R.M1	L.PMC-L.M1	R.PMC-L.PMC	
R.SMA-L.SMA	L.SMA-L.M1	L.SMA-R.M1	R.Thal-L.SMA	L.SMA-R.M1	R.Thal-R.SMA	L.Thal-L.SMA	R.SMA-L.PMC	
	L.PMC-R.M1	R.SMA-L.SMA		L.SMA-L.PMC		R.Thal-L.Thal		
	R.Thal-R.PMC							
**9HPT(A)**	**9HPT(U)**	**ARAT(A)**	**SIS(ADL)**	**SIS(HF)**	**SIS(Mob)**	**SIS(PS)**	**BI**	**Outcomes at T7 Input rs-FC Features at T4**
L.SMA-R.M1	L.SMA-L.M1	R.PMC-L.M1	R.SMA-L.M1	L.PMC-L.M1	R.PMC-R.M1	R.SMA-R.M1	L.PMC-L.M1	
R.SMA-L.SMA	L.PMC-R.M1	R.SMA-R.M1	L.Thal-L.SMA	R.SMA-R.PMC	R.Thal-L.SMA	R.Thal-L.SMA	R.Thal-L.M1	
	L.Thal-R.M1	L.Thal-R.M1				R.Thal-L.Thal	L.SMA-L.PMC	
							R.SMA-L.PMC	
							R.SMA-R.PMC	
**9HPT(A)**	**9HPT(U)**	**ARAT(A)**	**SIS(ADL)**	**SIS(HF)**	**SIS(Mob)**	**SIS(PS)**	**BI**	**Outcomes at T7 Input rs-FC Features at T6**
R.PMC-R.M1	L.Thal-R.PMC	L.Thal-L.M1	R.M1-L.M1	R.PMC-R.M1	R.M1-L.M1	R.M1-L.M1	R.M1-L.M1	
R.Thal-L.PMC	L.Thal-L.SMA	R.PMC-R.M1	L.SMA-R.PMC	R.Thal-L.PMC	L.PMC-L.M1	R.PMC-L.M1	R.SMA-R.M1	
		R.Thal-R.PMC	L.Thal-R.PMC		L.SMA-L.PMC	R.SMA-R.M1	R.Thal-R.PMC	

						R.Thal-R.M1		

**(B) With Clinical Variables**								
**9HPT(A)**	**9HPT(U)**	**ARAT(A)**	**SIS(ADL)**	**SIS(HF)**	**SIS(Mob)**	**SIS(PS)**	**BI**	**Outcomes at T6 Input rs-FC and Clinical Features at T4**
Motor Imp.	R.M1-L.M1	R.M1-L.M1	R.Thal-L.M1	L.SMA-L.PMC	L.SMA-L.M1	R.PMC-R.M1	R.SMA-L.PMC	
TSS	L.SMA-L.M1	Motor Imp.	L.PMC-R.M1	Motor Imp.	L.PMC-R.M1	Lesion Hemi	Motor Imp.	
NIHSS	L.PMC-R.M1	TSS	R.Thal-L.SMA	TSS	R.PMC-R.M1	NIHSS	TSS	
	R.Thal-R.PMC		Lesion Hemi				NIHSS	
			Gender					
**9HPT(A)**	**9HPT(U)**	**ARAT(A)**	**SIS(ADL)**	**SIS(HF)**	**SIS(Mob)**	**SIS(PS)**	**BI**	**Outcomes at T7 Input rs-FC and Clinical Features at T4**
Motor Imp.	Lesion Hemi	L.PMC-L.M1	R.M1-L.M1	R.SMA-L.M1	L.PMC-L.M1	R.SMA-L.SMA	R.SMA-L.M1	
TSS	Motor Imp.	L.PMC-R.M1	Motor Imp.	L.Thal-L.SMA		Motor Imp.	R.SMA-R.M1	
NIHSS		Age	NIHSS				R.SMA-R.PMC	
							Motor Imp.	
							TSS	
**9HPT(A)**	**9HPT(U)**	**ARAT(A)**	**SIS(ADL)**	**SIS(HF)**	**SIS(Mob)**	**SIS(PS)**	**BI**	**Outcomes at T7 Input rs-FC and Clinical Features at T6**
Motor Imp.	Motor Imp.	R.Thal-L.PMC	R.M1-L.M1	L.SMA-R.M1	R.M1-L.M1	R.SMA-R.M1	L.SMA-R.M1	
			L.SMA-R.PMC	R.Thal-L.PMC	R.Thal-L.M1	L.Thal-R.M1	R.SMA-L.PMC	
			NIHSS	Motor Imp.	L.PMC-R.M1	NIHSS	L.Thal-L.PMC	
					Lesion Hemi		Motor Imp.	

ST 5. List of Δrs-FC correlates of Δbehavior between all pairs of time-points from non-linear SVR are presented below.

**(A) Without Clinical Variables**								
**Δ9HPT(A)**	**Δ9HPT(U)**	**ΔSIS(ADL)**	**ΔSIS(Mob)**	**ΔSIS(PS)**	**ΔBI**	**ΔARAT(A)**	**ΔSIS(HF)**	**Outcome at T6 Input Δrs-FC Features at T4**
L.SMA-L.M1	R.SMA-L.M1	R.PMC-R.M1	L.PMC-R.M1	R.PMC-L.M1	R.Thal-L.M1	R.SMA-R.M1	R.SMA-L.M1	
R.SMA-L.M1	R.Thal-L.SMA	L.Thal-R.M1	R.PMC-R.M1	L.SMA-L.M1	R.Thal-R.M1	R.Thal-R.M1	R.SMA-R.M1	
L.PMC-R.M1	R.Thal-R.SMA	R.PMC-L.PMC	L.Thal-L.SMA	L.SMA-L.PMC		L.Thal-L.SMA	R.Thal-R.M1	
R.SMA-L.PMC				R.SMA-L.PMC		R.Thal-R.SMA	R.Thal-L.Thal	
R.Thal-L.Thal				R.Thal-R.PMC				
				R.Thal-L.SMA				
**Δ9HPT(A)**	**Δ9HPT(U)**	**ΔSIS(ADL)**	**ΔSIS(Mob)**	**ΔSIS(PS)**	**ΔBI**	**ΔARAT(A)**	**ΔSIS(HF)**	**Outcome at T7 Input Δrs-FC Features at T4**
R.M1-L.M1	L.PMC-R.M1	L.SMA-L.M1	L.SMA-R.M1	L.SMA-L.M1	L.PMC-R.M1	L.Thal-L.PMC	R.SMA-L.M1	
R.PMC-L.M1	L.SMA-R.PMC	R.SMA-L.M1	L.Thal-R.M1	L.Thal-R.SMA	R.SMA-R.M1	R.SMA-R.PMC	L.PMC-R.M1	
L.Thal-L.M1	L.Thal-R.PMC	R.PMC-R.M1	R.PMC-L.PMC		L.Thal-R.SMA		R.SMA-L.PMC	
R.PMC-R.M1	L.Thal-R.SMA	L.SMA-R.M1	R.SMA-L.PMC				L.Thal-L.PMC	
R.Thal-R.M1		R.SMA-R.M1	R.SMA-R.PMC				R.Thal-L.PMC	
R.SMA-R.PMC		L.SMA-L.PMC	L.Thal-L.SMA				R.Thal-R.PMC	
L.Thal-R.PMC		R.SMA-L.PMC	R.Thal-L.Thal					
R.Thal-R.SMA		R.SMA-L.SMA						
		R.Thal-L.SMA						
		L.Thal-R.SMA						
		R.Thal-R.SMA						
**Δ9HPT(A)**	**Δ9HPT(U)**	**ΔSIS(ADL)**	**ΔSIS(Mob)**	**ΔSIS(PS)**	**ΔBI**	**ΔARAT(A)**	**ΔSIS(HF)**	**Outcome at T7 Input Δrs-FC Features at T6**
L.PMC-R.M1	R.Thal-R.M1	R.M1-L.M1	L.SMA-L.M1	R.M1-L.M1	R.PMC-L.M1	R.PMC-L.M1	L.SMA-L.M1	
L.SMA-R.PMC	L.SMA-L.PMC	L.PMC-L.M1	L.SMA-R.M1	L.Thal-L.M1	R.PMC-L.PMC	R.Thal-R.M1		
R.SMA-R.PMC	R.Thal-L.Thal	L.Thal-L.PMC	R.Thal-L.SMA	R.SMA-R.PMC		R.PMC-L.PMC		
		R.SMA-R.PMC		R.Thal-R.PMC				
		R.Thal-R.PMC						
		R.Thal-R.SMA						

(B) With Clinical Variables								
**Δ9HPT(A)**	**Δ9HPT(U)**	**ΔSIS(ADL)**	**ΔSIS(Mob)**	**ΔSIS(PS)**	**ΔBI**	**ΔARAT(A)**	**ΔSIS(HF)**	**Outcome at T6 Input Δrs-FC at T4 + Clinical Features**
R.SMA-L.M1	R.SMA-L.M1	R.PMC-R.M1	L.Thal-L.M1	L.SMA-L.M1	R.Thal-L.M1	R.SMA-R.M1	R.PMC-L.M1	
R.SMA-L.SMA	R.PMC-L.PMC	L.Thal-R.M1	L.Thal-R.PMC	L.Thal-R.M1	L.Thal-L.SMA	R.Thal-R.M1	R.Thal-L.SMA	
Gender	R.Thal-R.SMA	TSS	R.Thal-L.SMA	L.SMA-L.PMC	R.Thal-R.SMA	R.Thal-R.SMA		
				R.Thal-R.PMC				
				R.Thal-L.SMA				
				NIHSS				
**Δ9HPT(A)**	**Δ9HPT(U)**	**ΔSIS(ADL)**	**ΔSIS(Mob)**	**ΔSIS(PS)**	**ΔBI**	**ΔARAT(A)**	**ΔSIS(HF)**	**Outcome at T7 Input Δrs-FC at T4 + Clinical Features**
R.PMC-L.PMC	R.Thal-R.M1	R.SMA-L.M1	R.PMC-L.M1	L.SMA-L.M1	R.SMA-R.M1	L.SMA-L.M1	R.PMC-L.M1	
R.SMA-R.PMC	L.SMA-R.PMC	R.PMC-R.M1	L.SMA-R.M1	L.PMC-R.M1	L.SMA-R.PMC	R.Thal-R.SMA	L.SMA-L.M1	
R.SMA-L.SMA	R.SMA-L.SMA	R.SMA-L.PMC	R.SMA-L.SMA	L.Thal-L.SMA	L.Thal-R.SMA	TSS	L.Thal-R.M1	
	Motor Imp.	R.Thal-L.SMA	L.Thal-L.SMA	L.Thal-R.SMA	Motor Imp.		L.Thal-R.PMC	
			Motor Imp.	Age	NIHSS		R.Thal-R.PMC	
				NIHSS			R.SMA-L.SMA	
**Δ9HPT(A)**	**Δ9HPT(U)**	**ΔSIS(ADL)**	**ΔSIS(Mob)**	**ΔSIS(PS)**	**ΔBI**	**ΔARAT(A)**	**ΔSIS(HF)**	**Outcome at T7 Input Δrs-FC at T6 + Clinical Features**
R.M1-L.M1	R.SMA-L.SMA	L.PMC-L.M1	L.SMA-L.M1	R.M1-L.M1	R.PMC-L.M1	R.PMC-L.M1	L.SMA-L.M1	
R.SMA-L.M1	R.Thal-L.Thal	L.SMA-R.M1	R.Thal-L.SMA	L.Thal-L.M1	R.PMC-L.PMC	R.Thal-R.M1		
L.PMC-R.M1		L.Thal-L.PMC	Motor Imp.	R.SMA-R.PMC		R.PMC-L.PMC		
R.SMA-L.SMA		R.Thal-R.PMC		R.Thal-R.PMC		Age		
				Lesion Hemi		TSS		
				NIHSS		Gender		
						NIHSS		

ST 6. List of behavioral correlates at preceding time-points to for estimation of measures at succeeding time-points from non-linear SVR are presented below.

**9HPT(A)**	**9HPT(U)**	**ARAT(A)**	**SIS(ADL)**	**SIS(HF)**	**SIS(Mob)**	**SIS(PS)**	**BI**	**Outcomes at T6 Input Behavior at T4 + Clinical Variables**
9HPT(A)	9HPT(U)	ARAT(A)	SIS(ADL)	SIS(HF)	SIS(Mob)	Lesion Hemi	BI	
Lesion Hemi	Gender	Motor Imp.	Motor Imp.	NIHSS	Lesion Hemi	NIHSS		
		NIHSS						
**9HPT(A)**	**9HPT(U)**	**ARAT(A)**	**SIS(ADL)**	**SIS(HF)**	**SIS(Mob)**	**SIS(PS)**	**BI**	**Outcomes at T7 Input Behavior at T4 + Clinical Variables**
9HPT(A)	9HPT(U)	ARAT(A)	SIS(ADL)	SIS(HF)	SIS(Mob)	Motor Imp.	BI	
		Lesion Hemi	Motor Imp.	NIHSS	Gender		Motor Imp.	
		Motor Imp.	Gender				TSS	
**9HPT(A)**	**9HPT(U)**	**ARAT(A)**	**SIS(ADL)**	**SIS(HF)**	**SIS(Mob)**	**SIS(PS)**	**BI**	**Outcomes at T7 Input Behavior at T6 + Clinical Variables**
9HPT(A)	9HPT(U)	ARAT(A)	SIS(ADL)	SIS(HF)	SIS(Mob)	SIS(PS)	BI	
Motor Imp.		Lesion Hemi			NIHSS	Lesion Hemi	TSS	
							NIHSS	

2. Permutation tests

For permutation tests, the following legend is followed for all images:

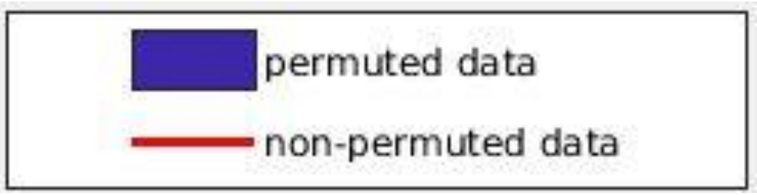

When the RMSE from the non-permuted data is lower than 95% of null distribution from permuted data, the non-permuted model is said to be performing significantly better than chance-level (p<0.05).

SF 3(A). Permutation test performed to test SVR models against chance level. Plots are for the following outcomes: (a) 9HPT(A); (b) 9HPT(U); (c) ARAT(A); (d) SIS(ADL); (e) SIS(HF); (f) SIS(Mob); (g) SIS(PS) (h) BI.

Linear SVR: **Input:** rs-FC at pre-intervention; **Outcome:** behavioral outcomes at post-intervention.

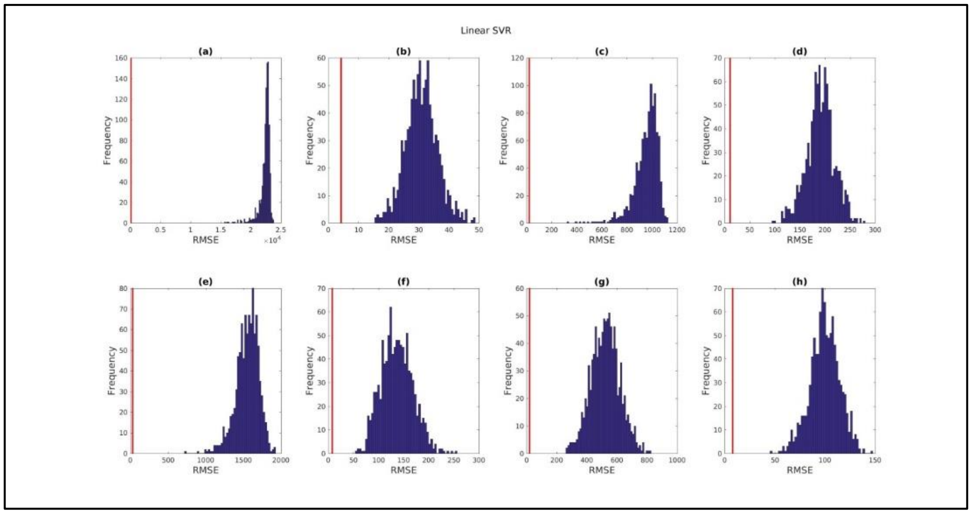

Linear SVR: **Input:** rs-FC at pre-intervention and clinical variables; **Outcome:** behavioral outcomes at post-intervention.

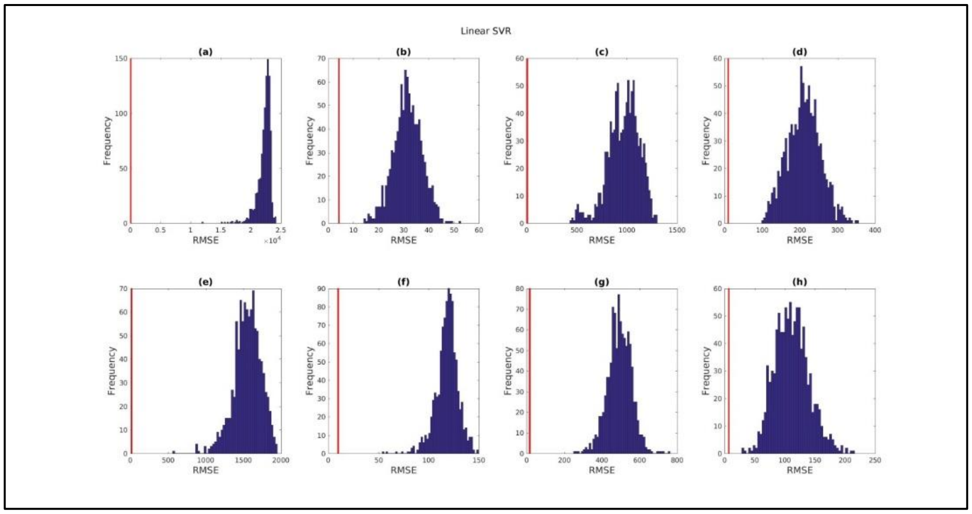

Non-linear SVR: **Input:** rs-FC at pre-intervention; **Outcome:** behavioral outcomes at post-intervention.

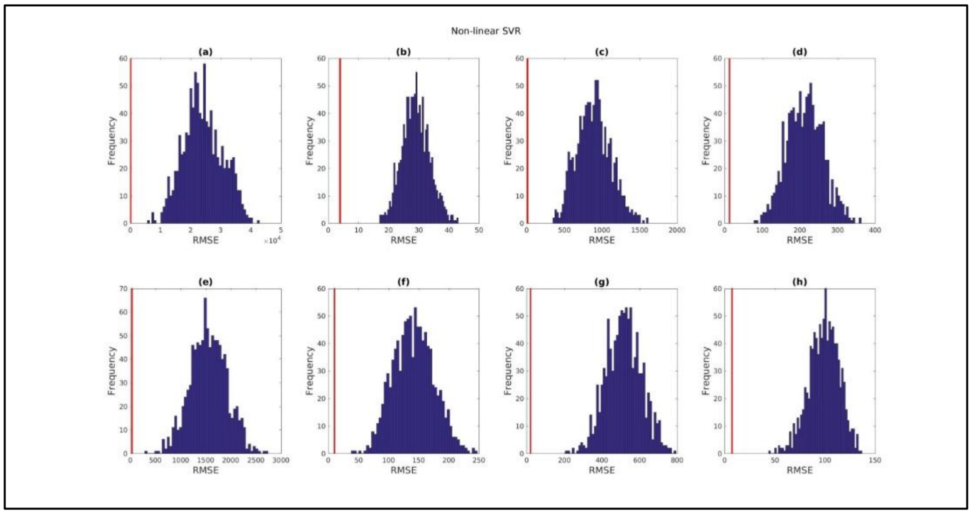

Non-linear SVR: **Input:** rs-FC at pre-intervention and clinical variables; **Outcome:** behavioral outcomes at post-intervention.

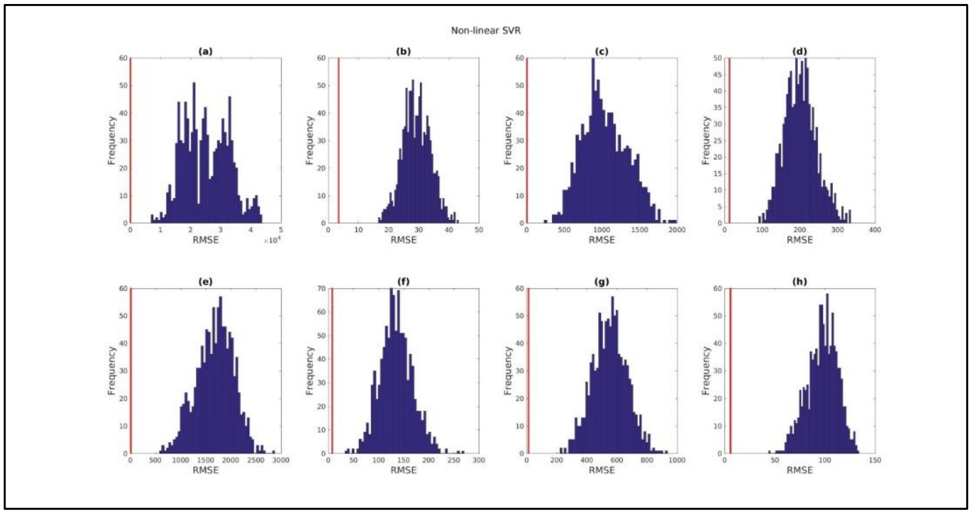

SF 3(B). Permutation test performed to test SVR models against chance level. Plots are for the following outcomes: (a) 9HPT(A); (b) 9HPT(U); (c) ARAT(A); (d) SIS(ADL); (e) SIS(HF); (f) SIS(Mob); (g) SIS(PS) (h) BI.

Linear SVR: **Input:** rs-FC at pre-intervention; **Outcome:** behavioral outcomes at one-month post-intervention.

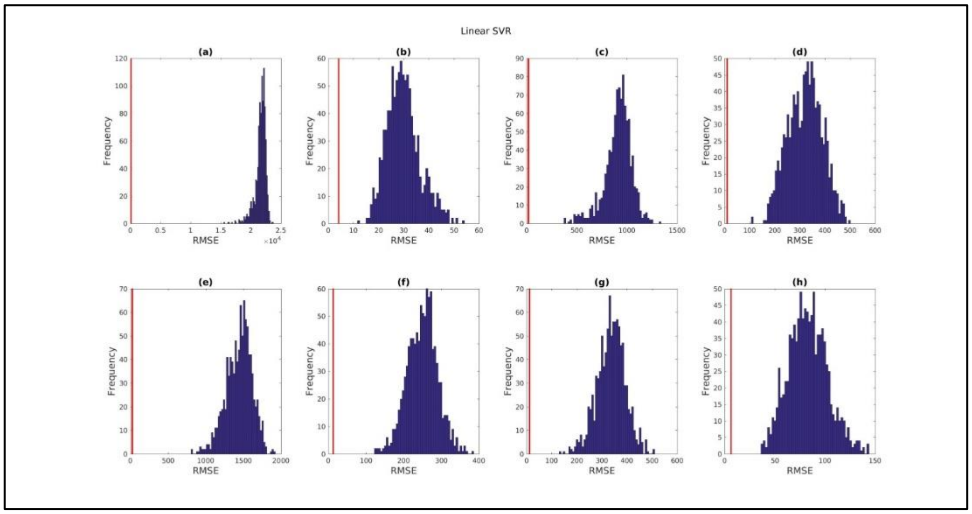

Linear SVR: **Input:** rs-FC at pre-intervention and clinical variables; **Outcome:** behavioral outcomes at one-month post-intervention.

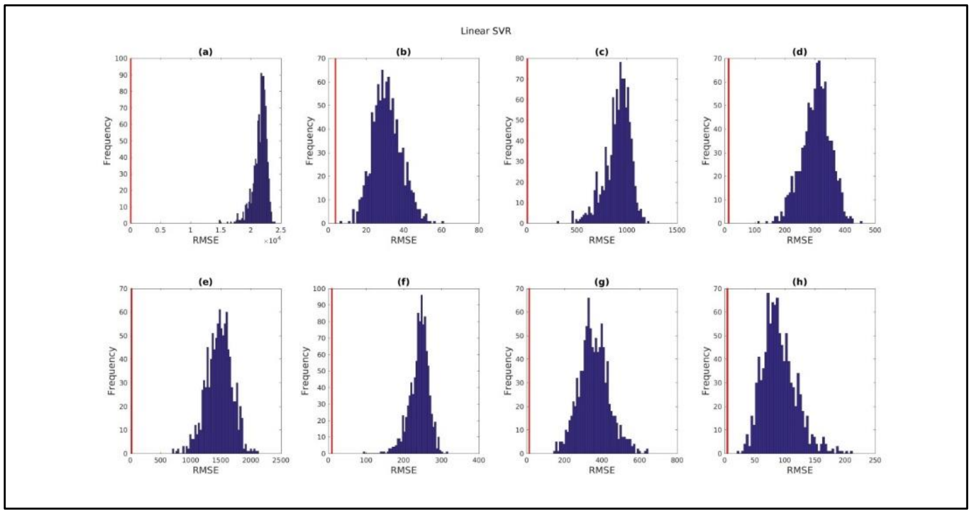

Non-linear SVR: **Input:** rs-FC at pre-intervention; **Outcome:** behavioral outcomes at one-month post-intervention.

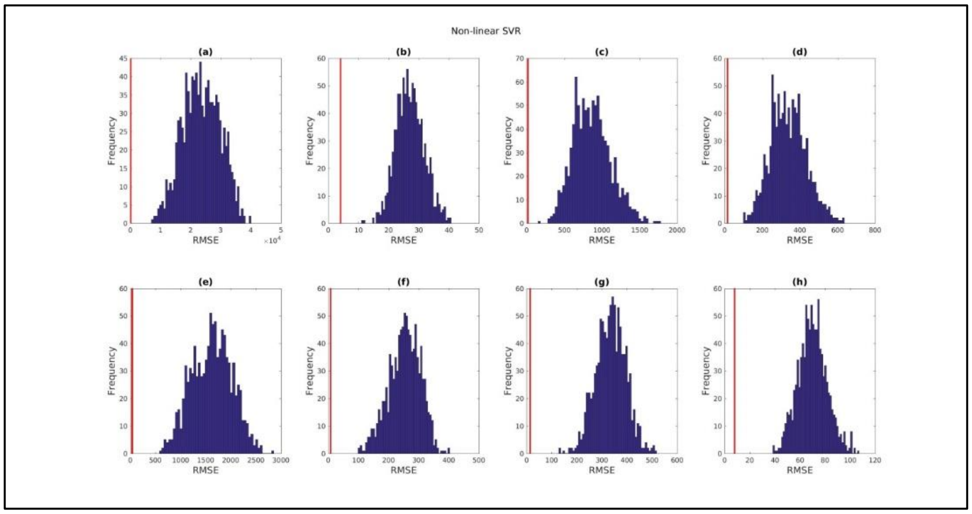

Non-linear SVR: **Input:** rs-FC at pre-intervention and clinical variables; **Outcome:** behavioral outcomes at one-month post-intervention.

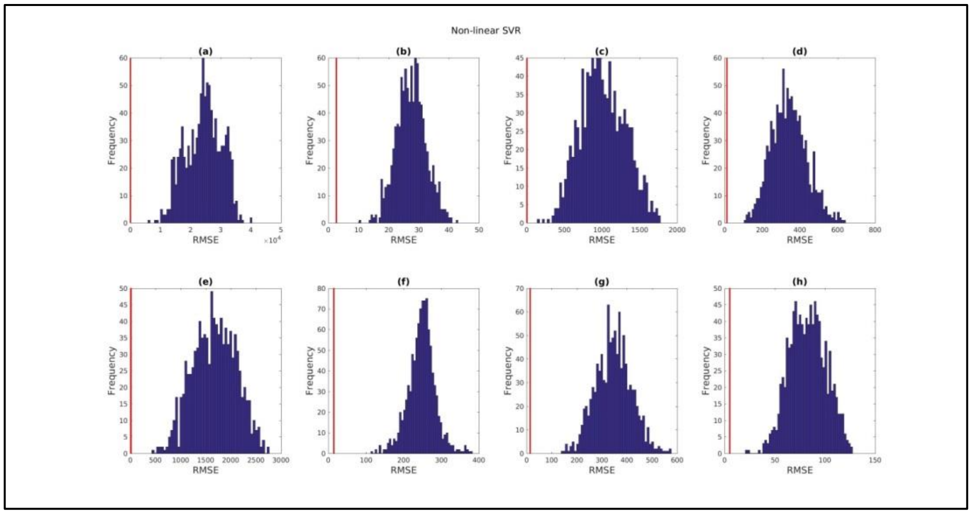

SF 3(C). Permutation test performed to test SVR models against chance level. Plots are for the following outcomes: (a) 9HPT(A); (b) 9HPT(U); (c) ARAT(A); (d) SIS(ADL); (e) SIS(HF); (f) SIS(Mob); (g) SIS(PS) (h) BI.

Linear SVR: **Input:** rs-FC at post-intervention; **Outcome:** behavioral outcomes at one-month post-intervention.

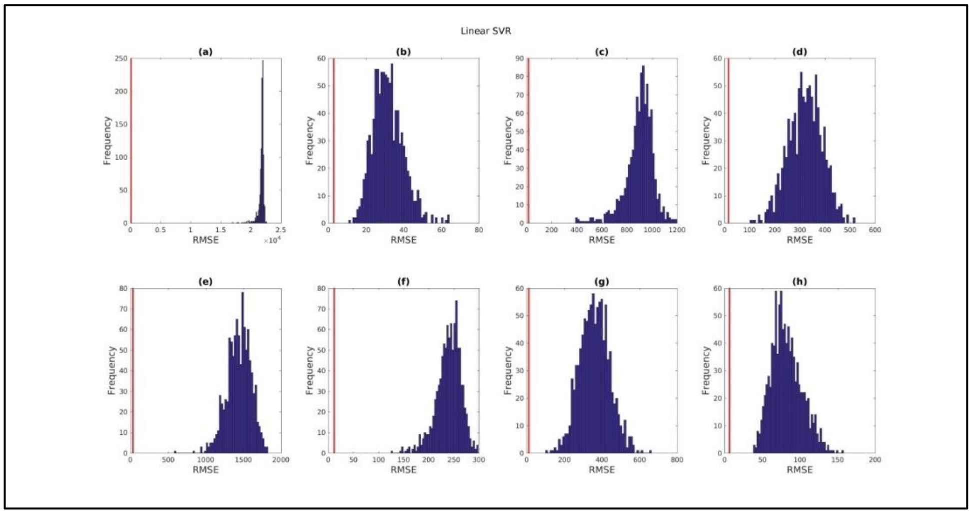

Linear SVR: **Input:** rs-FC at post-intervention and clinical variables; **Outcome:** behavioral outcomes at one-month post-intervention.

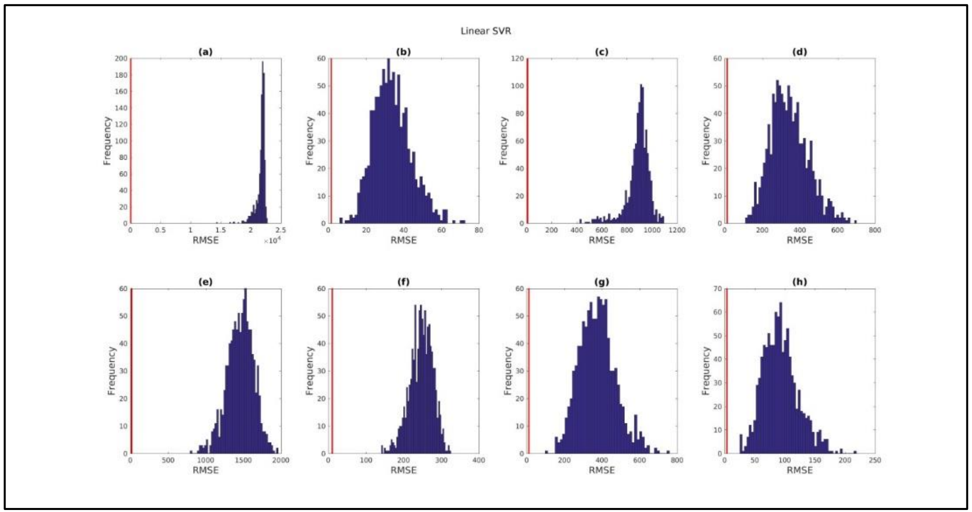

Non-linear SVR: **Input:** rs-FC at post-intervention; **Outcome:** behavioral outcomes at one-month post-intervention.

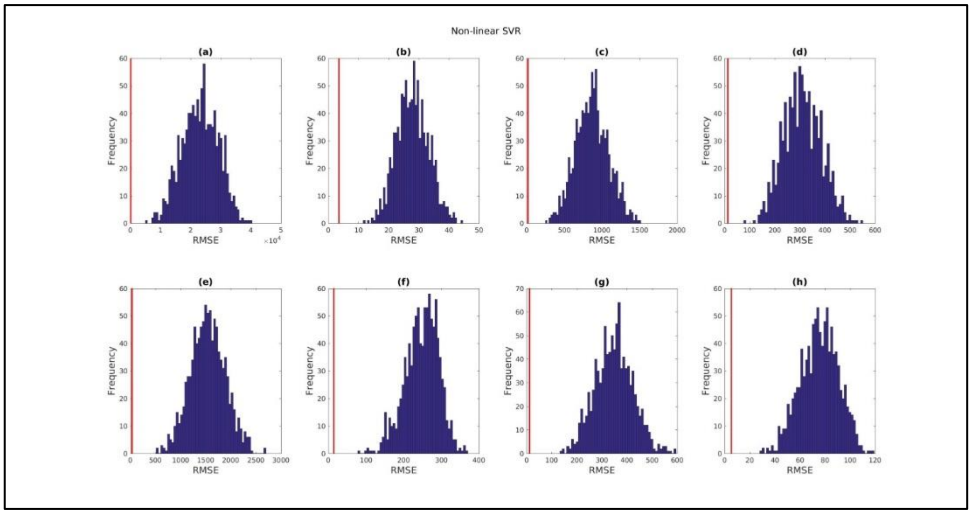

Non-linear SVR: **Input:** rs-FC at post-intervention and clinical variables; **Outcome:** behavioral outcomes at one-month post-intervention.

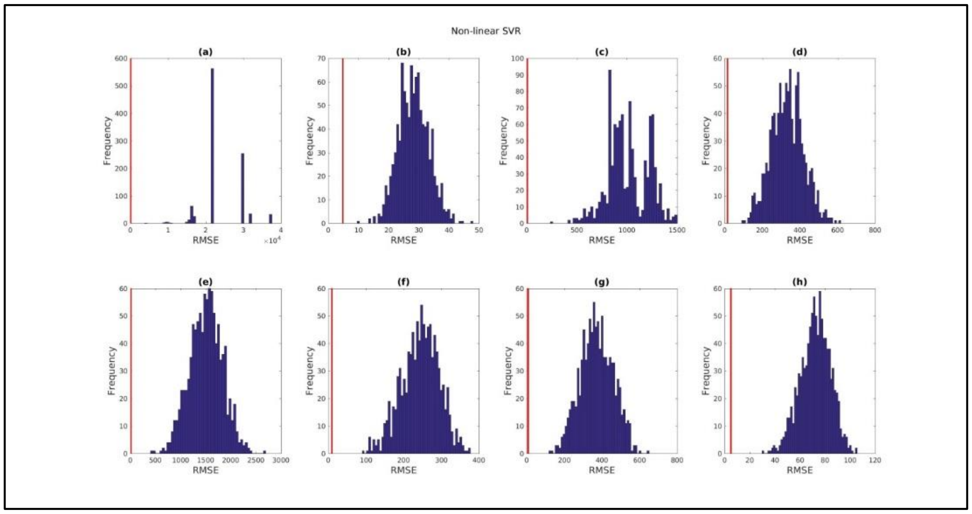

SF 4(A). Permutation test performed to test SVR models against chance level. Plots are for the following outcomes: (a) 9HPT(A); (b) 9HPT(U); (c) SIS(ADL); (d) SIS(Mob); (e) SIS(PS); (f) BI; (g) ARAT(A); (h) SIS(HF).

Linear SVR: **Input:** Δ rs-FC between at pre-intervention and post-intervention; **Outcome:** Δ behavior between pre-intervention and post-intervention.

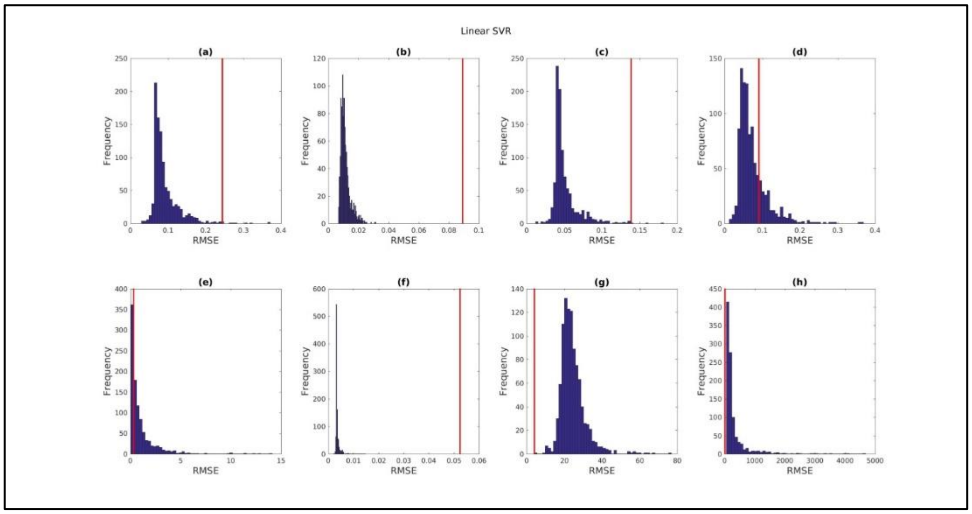

Linear SVR: **Input:** Δ rs-FC between at pre-intervention and post-intervention and clinical variables; **Outcome:** Δ behavior between pre-intervention and post-intervention.

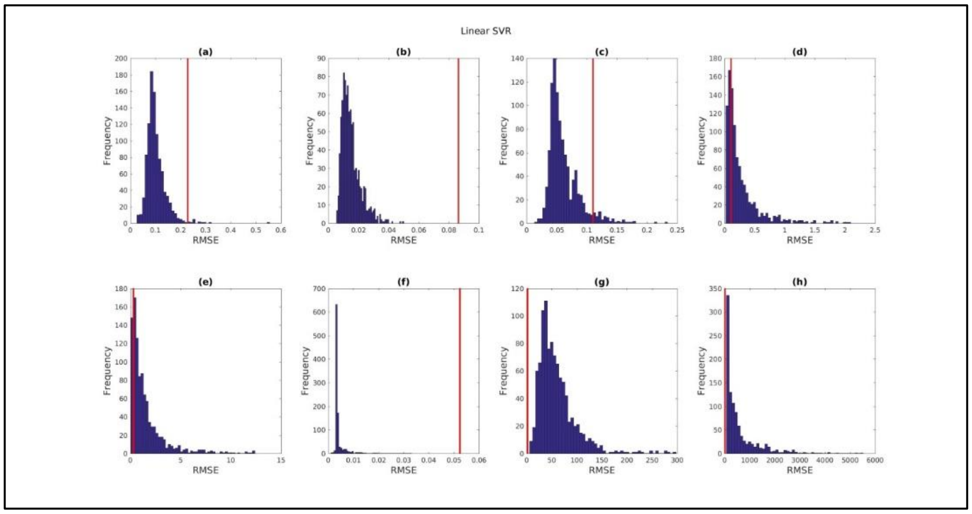

Non-linear SVR: **Input:** Δ rs-FC between at pre-intervention and post-intervention; **Outcome:** Δ behavior between pre-intervention and post-intervention.

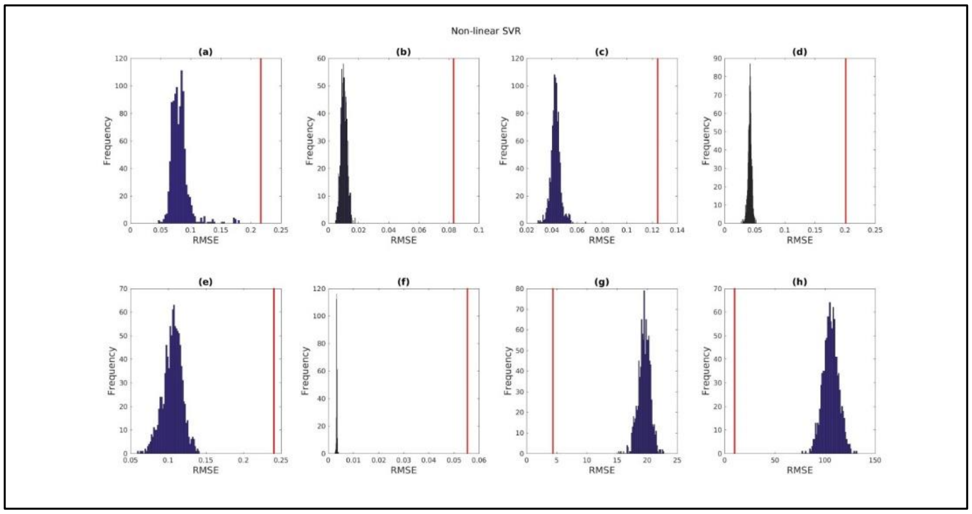

Non-linear SVR: **Input:** Δ rs-FC between at pre-intervention and post-intervention and clinical variables; **Outcome:** Δ behavior between pre-intervention and post-intervention.

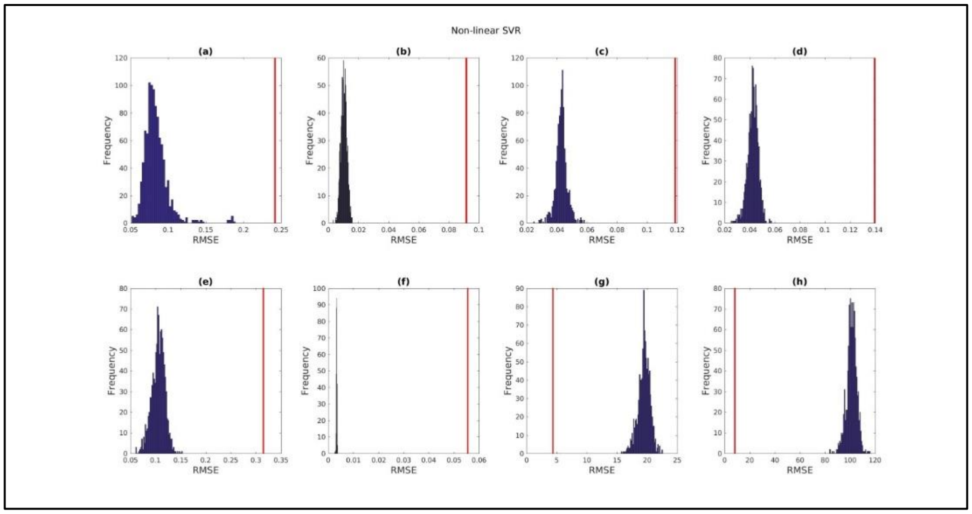

SF 4(B). Permutation test performed to test SVR models against chance level. Plots are for the following outcomes: (a) 9HPT(A); (b) 9HPT(U); (c) SIS(ADL); (d) SIS(Mob); (e) SIS(PS); (f) BI; (g) ARAT(A); (h) SIS(HF).

Linear SVR: **Input:** Δ rs-FC between post-intervention and one-month post-intervention; **Outcome:** Δ behavior between post-intervention and one-month post-intervention.

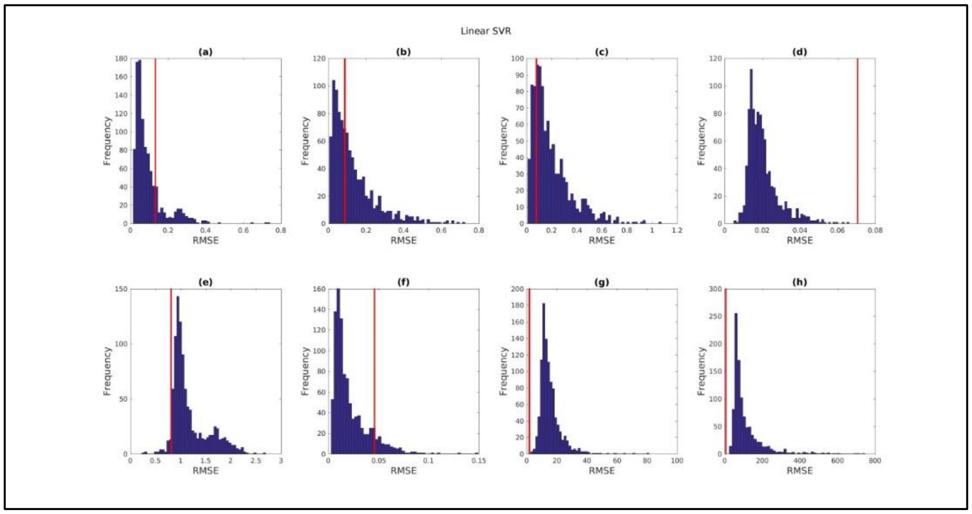

Linear SVR: **Input:** Δ rs-FC between post-intervention and one-month post-intervention and clinical variables; **Outcome:** Δ behavior changes between post-intervention and one-month post-intervention.

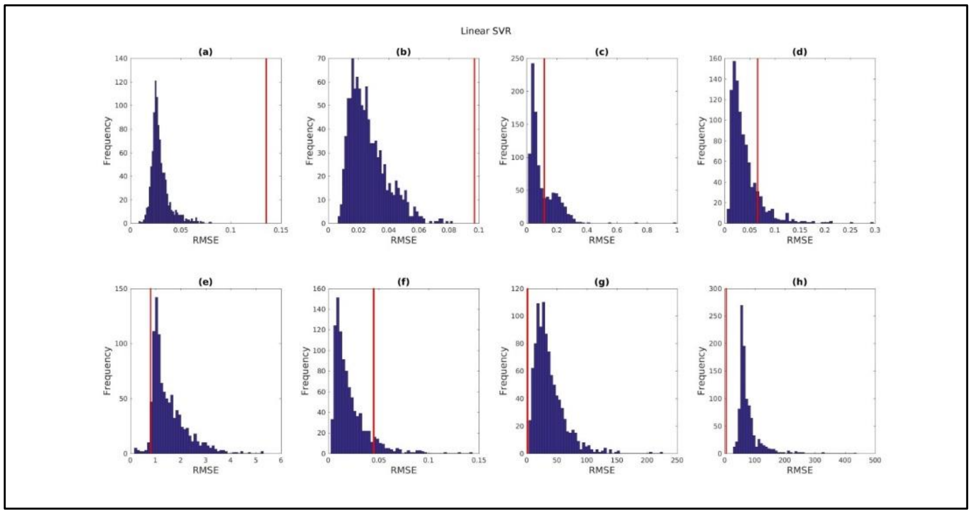

Non-linear SVR: **Input:** Δ rs-FC between post-intervention and one-month post-intervention; **Outcome:** Δ behavior between post-intervention and one-month post-intervention.

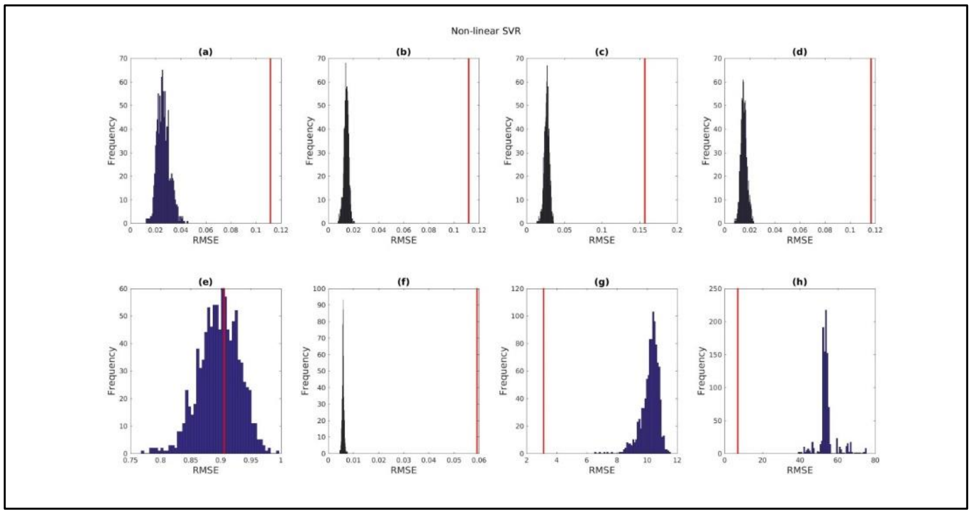

Non-linear SVR: **Input:** Δ rs-FC between post-intervention and one-month post-intervention and clinical variables; **Outcome:** Δ behavior between post-intervention and one-month post-intervention.

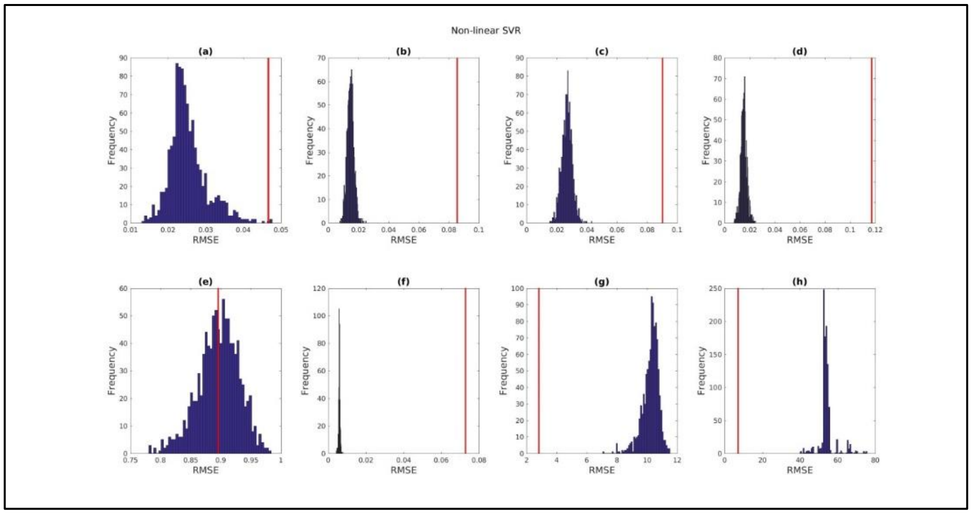

SF 4(C). Permutation test performed to test SVR models against chance level. Plots are for the following outcomes: (a) 9HPT(A); (b) 9HPT(U); (c) SIS(ADL); (d) SIS(Mob); (e) SIS(PS); (f) BI; (g) ARAT(A); (h) SIS(HF).

Linear SVR: **Input:** Δ rs-FC between at pre-intervention and one-month post-intervention; **Outcome:** Δ behavior between pre-intervention and one-month post-intervention.

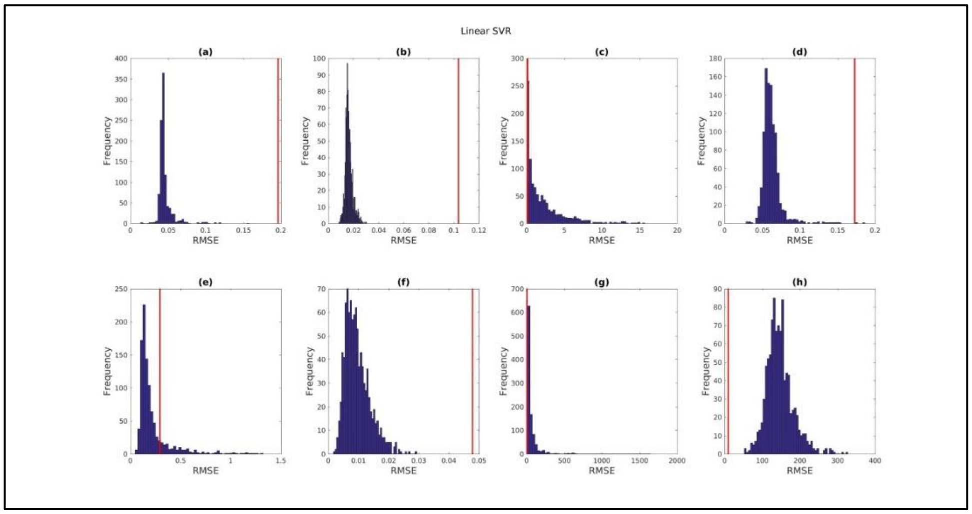

Linear SVR: **Input:** Δ rs-FC between at pre-intervention and one-month post-intervention and clinical variables; **Outcome:** Δ behavior between pre-intervention and one-month post-intervention.

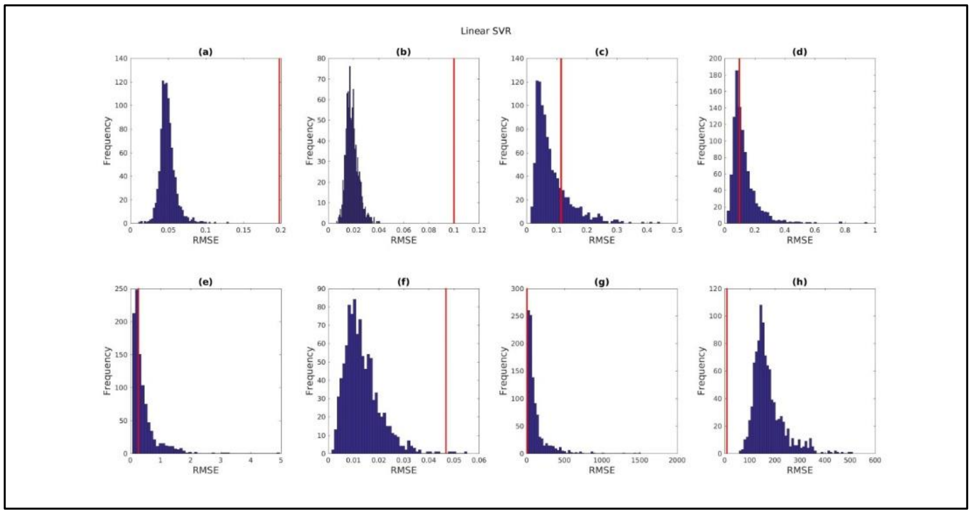

Non-linear SVR: **Input:** Δ rs-FC between at pre-intervention and one-month post-intervention; **Outcome:** Δ behavior between pre-intervention and one-month post-intervention.

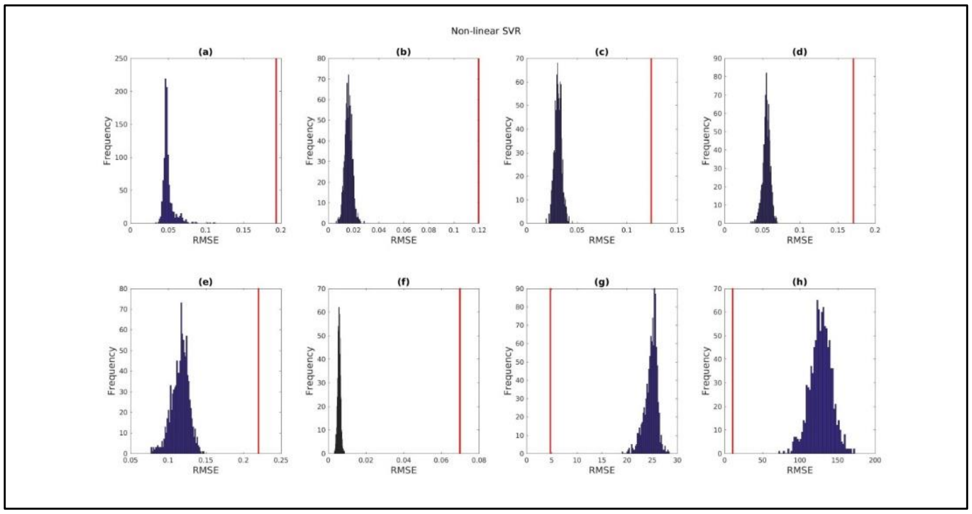

Non-linear SVR: **Input:** Δ rs-FC between at pre-intervention and one-month post-intervention and clinical variables; **Outcome:** Δ behavior between pre-intervention and one-month post-intervention.

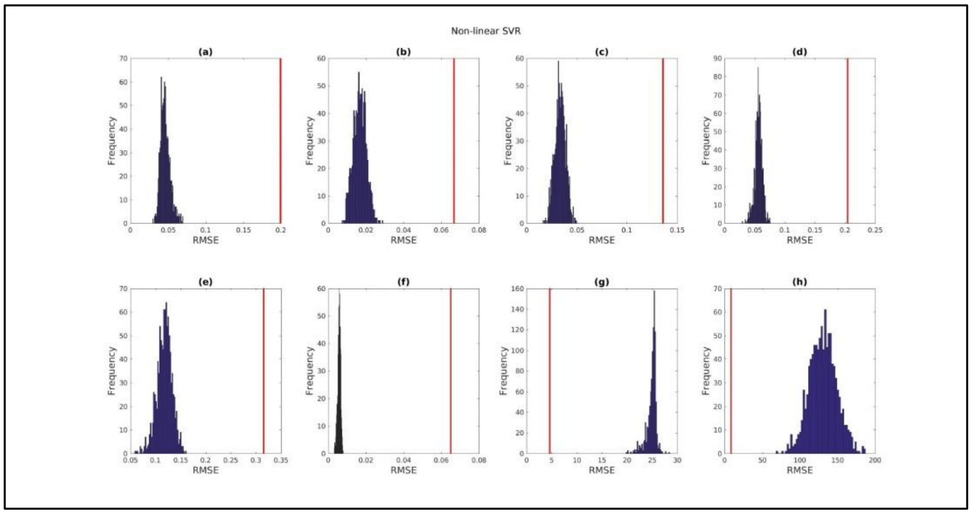

SF 5(A). Permutation test performed to test SVR models against chance level. **Input:** behavioral and clinical features at pre-intervention; **Outcome:** behavioral outcomes at post-intervention. Plots are for the following outcomes: (a) 9HPT(A); (b) 9HPT(U); (c) ARAT(A); (d) SIS(ADL); (e) SIS(HF); (f) SIS(Mob); (g) SIS(PS) (h) BI.

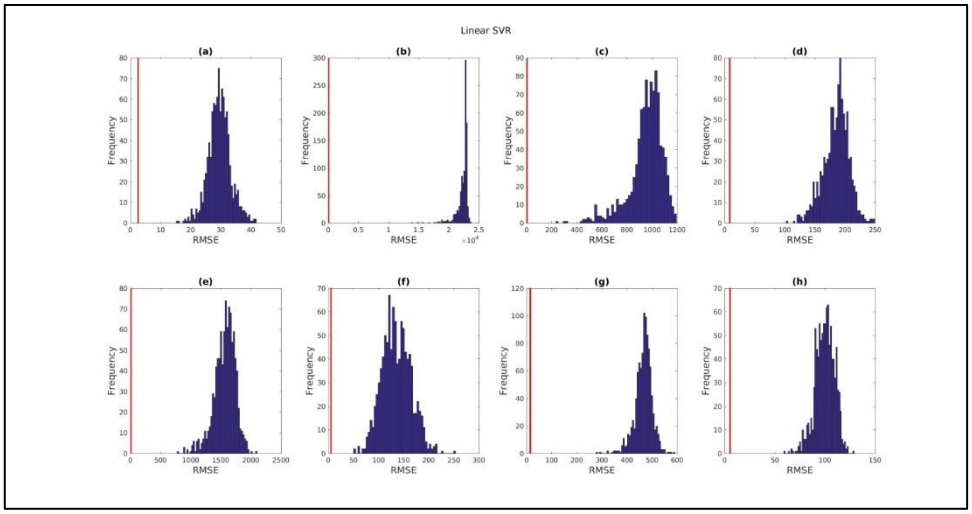

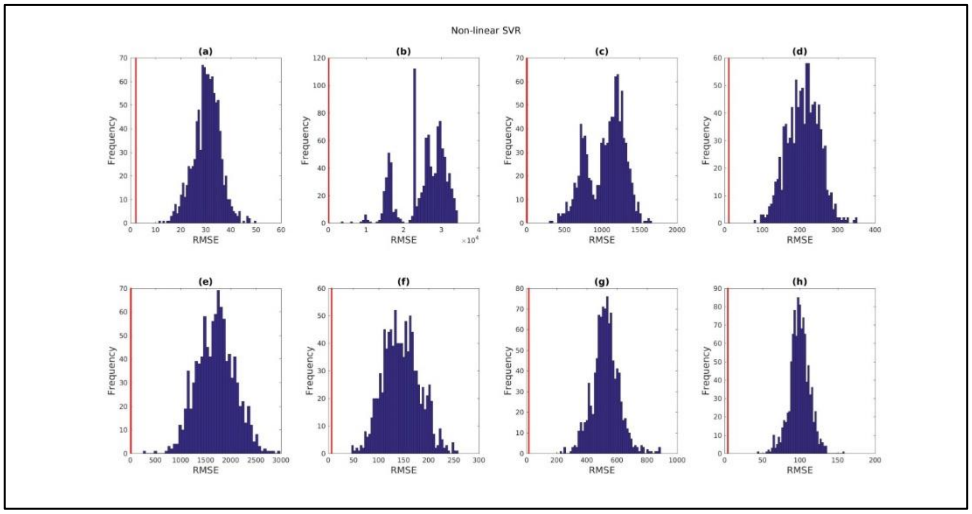

SF 5(B). Permutation test performed to test SVR models against chance level. **Input:** behavioral and clinical features at pre-intervention; **Outcome:** behavioral outcomes at one-month post-intervention. Plots are for the following outcomes: (a) 9HPT(A); (b) 9HPT(U); (c) ARAT(A); (d) SIS(ADL); (e) SIS(HF); (f) SIS(Mob); (g) SIS(PS) (h) BI.

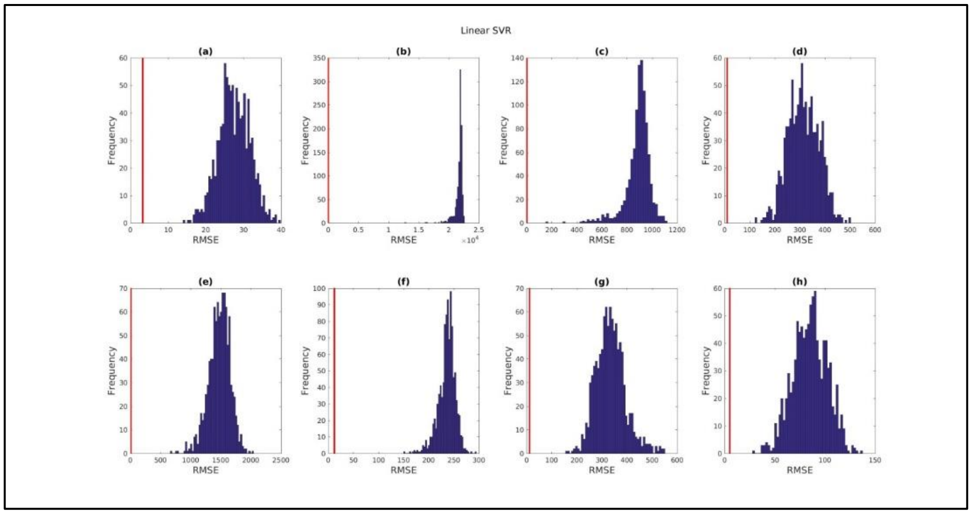

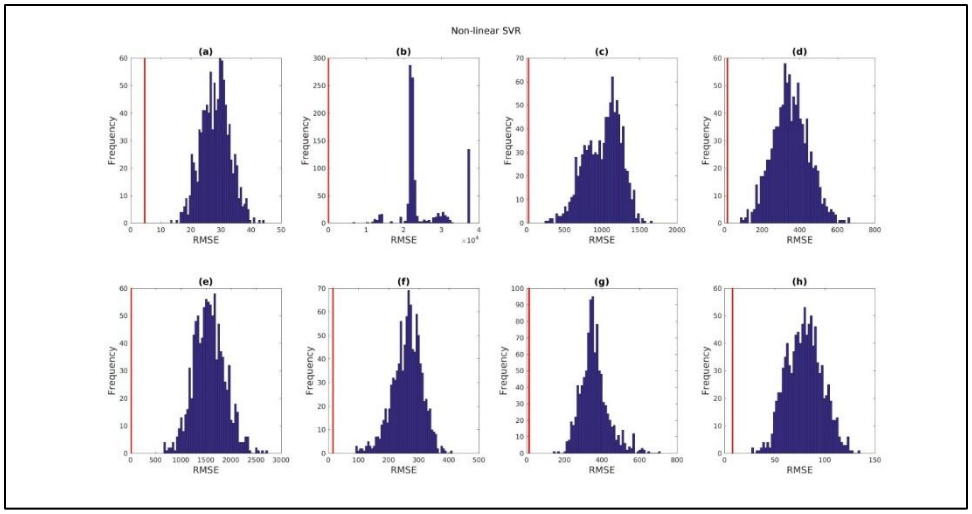

SF 5(C). Permutation test performed to test SVR models against chance level. **Input:** behavioral and clinical features at post-intervention; **Outcome:** behavioral outcomes at one-month post-intervention. Plots are for the following outcomes: (a) 9HPT(A); (b) 9HPT(U); (c) ARAT(A); (d) SIS(ADL); (e) SIS(HF); (f) SIS(Mob); (g) SIS(PS) (h) BI.

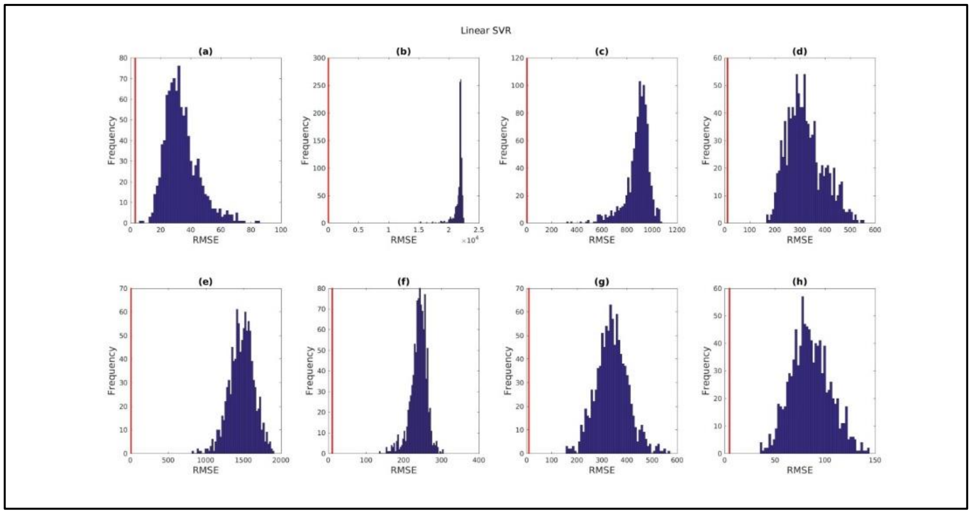

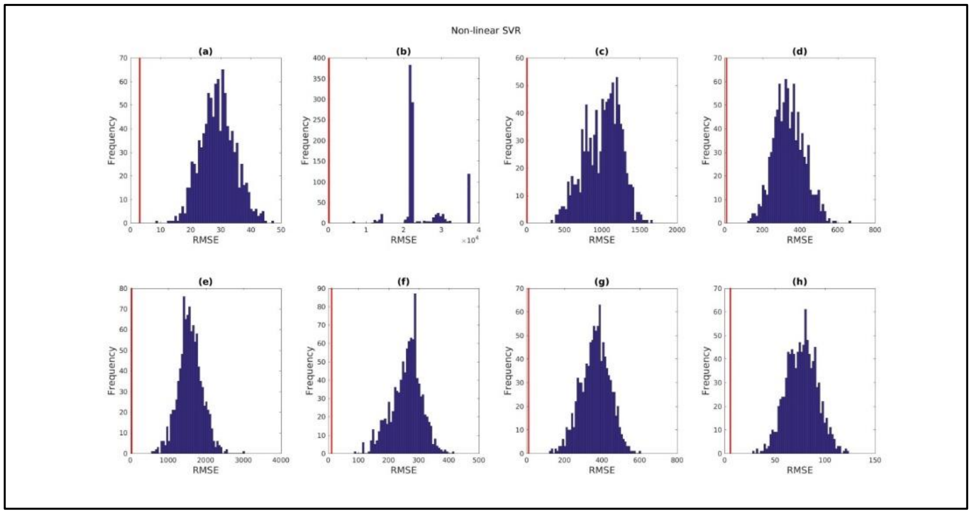

SF 6 (A). Comparison of RMSE between linear and non-linear SVR is shown for using rs-FC at preceding stages to estimate behavioral measures at succeeding stages: (a) Input: rs-FC at T4; Outcome: behavioral measures at T6; (b) Input: rs-FC at T4; Outcome: behavioral measures at T7;(c) Input: rs-FC at T6; Outcome: behavioral measures at T7.

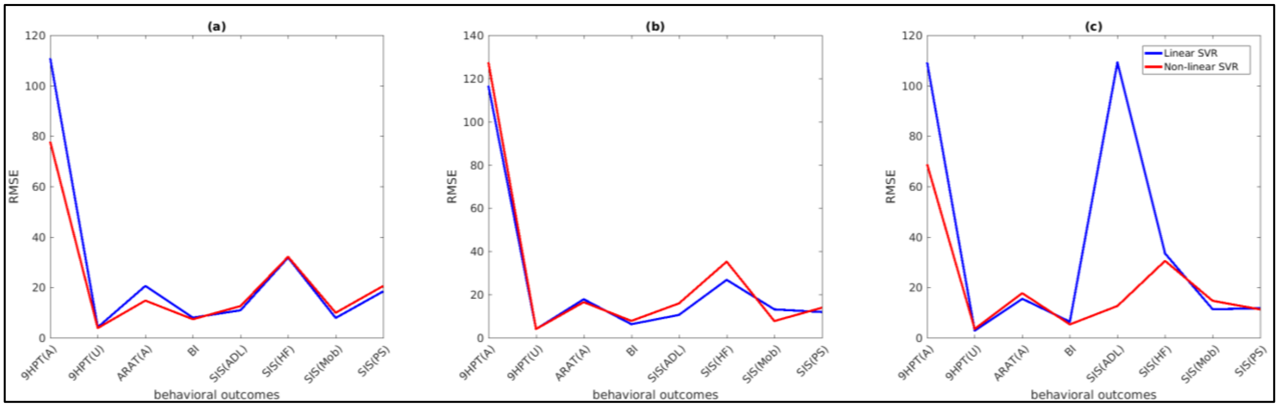

SF 6(B). Comparison of RMSE between linear and non-linear SVR is shown below for using rs-FC and clinical variables at preceding staged to estimate behavioral measures at succeeding stages: (a) Input: rs-FC at T4 and clinical variables; Outcome: behavioral measures at T6 ;(b) Input: rs-FC at T4 and clinical variables; Outcome: behavioral measures at T7;(c) Input: rs-FC at T6 and clinical variables; Outcome: behavioral measures at T7.

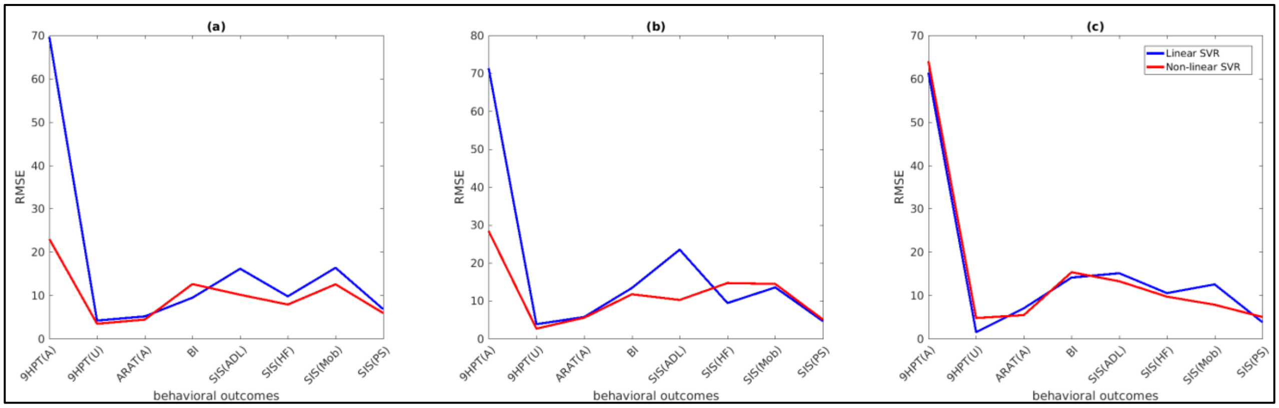

SF 7(A). Comparison of RMSE between linear and non-linear SVR is shown for using change in rs-FC between stages to estimate changes in behavioral measures between the corresponding stages: (a) Input: rs-FC changes between T4 and T6; Outcome: behavioral changes between T4 and T6 ; (b) Input: rs-FC changes between T4 and T7; Outcome: behavioral changes between T4 and T7; (c) Input: rs-FC changes between T6 and T7; Outcome: behavioral changes between T6 and T7.

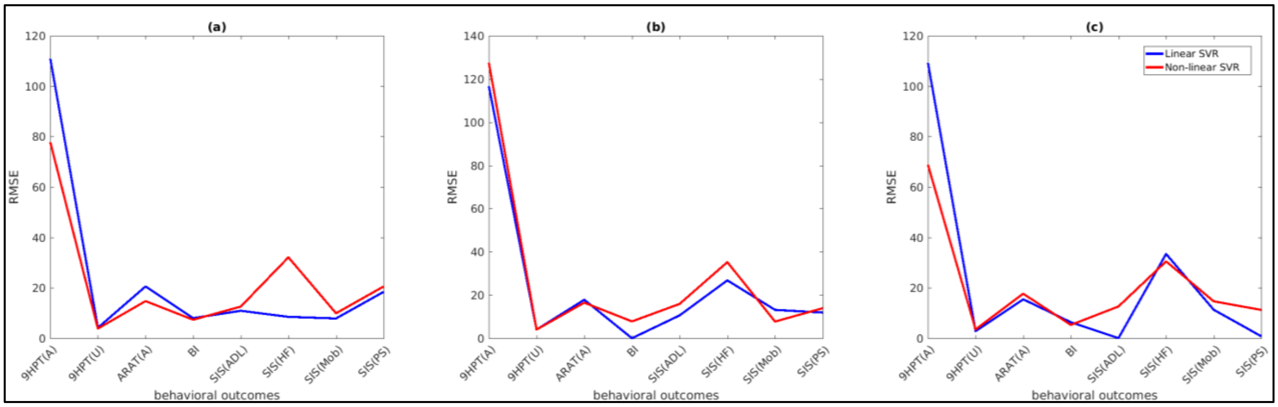

SF 7(B). Comparison of RMSE between linear and non-linear SVR is shown for using change in rs-FC between stages and clinical variables to estimate changes in behavioral measures between the corresponding stages: (a) Input: rs-FC changes between T4 and T6 and clinical variables; Outcome: behavioral changes between T4 and T6 and clinical variables; (b) Input: rs-FC changes between T4 and T7 and clinical variables; Outcome: behavioral changes between T4 and T7; (c) Input: rs-FC changes between T6 and T7 and clinical variables; Outcome: behavioral changes between T6 and T7.

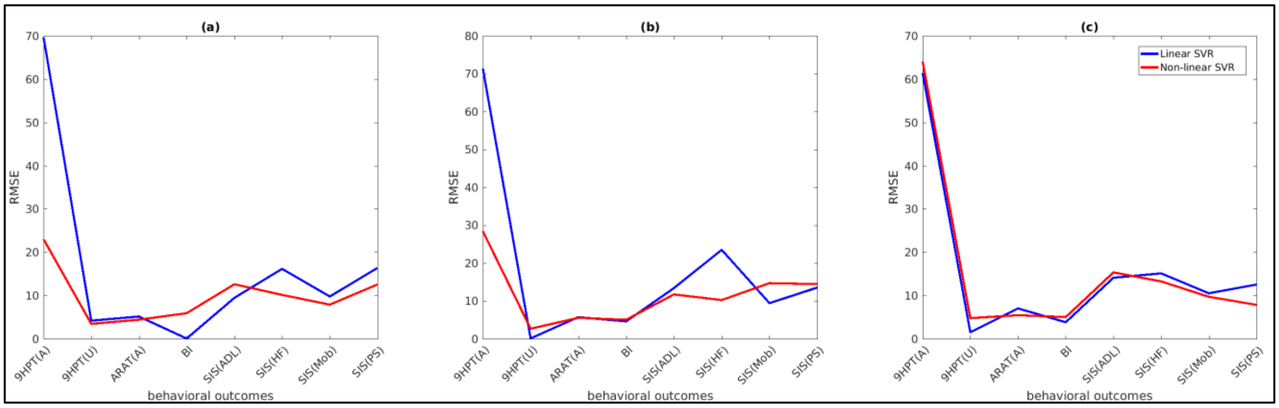

SF 8. Comparison of RMSE between linear and non-linear SVR is shown for using behavioral measures at preceding stages to estimate behavioral measures at succeeding stages: (a) Input: behavioral measures at T4; Outcome: behavioral measures at T6; (b) Input: behavioral measures at T4; Outcome: behavioral measures at T7; (c) Input: behavioral measures at T6; Outcome: behavioral measures at T7.

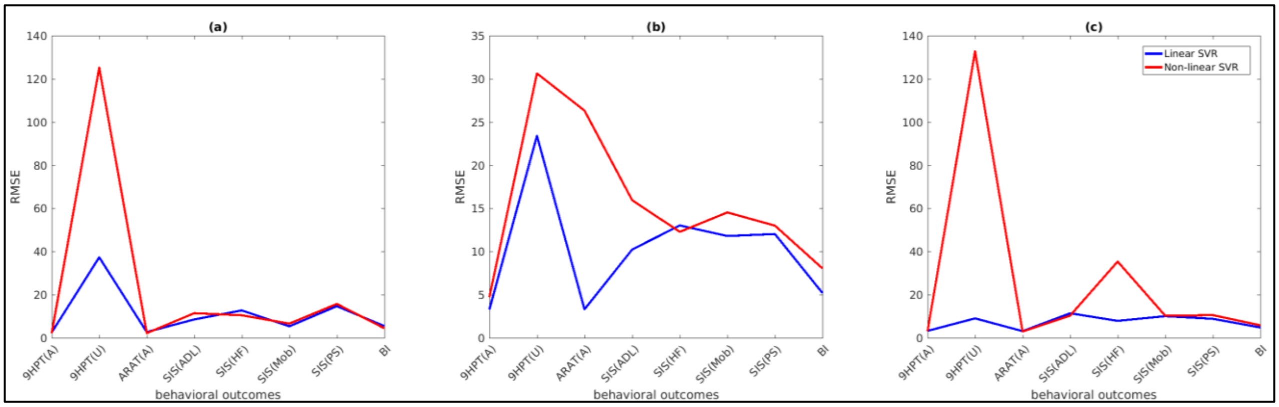

Supplement: Supplementary file 1 [file Data_Sheet_1.PDF]
